# Wintertime Air Quality across the Kathmandu Valley, Nepal: Concentration, Composition, and Sources of Fine and Coarse Particulate Matter

**DOI:** 10.1021/acsearthspacechem.2c00243

**Published:** 2022-12-06

**Authors:** Md. Robiul Islam, Tianyi Li, Khadak Mahata, Nita Khanal, Benjamin Werden, Michael R. Giordano, Siva Praveen Puppala, Narayan Babu Dhital, Anobha Gurung, Eri Saikawa, Arnico K. Panday, Robert J. Yokelson, Peter F. DeCarlo, Elizabeth. A. Stone

**Affiliations:** † Department of Chemistry, University of Iowa, Iowa City, Iowa 52242, United States; ‡ Alpine Consultancy, Kathmandu 44600, Nepal; § Department of Civil, Architectural, and Environmental Engineering, Drexel University, Philadelphia, Pennsylvania 19104, United States; ∥ Univ Paris Est Creteil and Université de Paris, CNRS, LISA, Créteil 94000, France; ⊥ International Centre for Integrated Mountain Development (ICIMOD), Khumaltar, Lalitpur 44700, Nepal; # Patan Multiple Campus, Department of Environmental Science, Tribhuvan University, Lalitpur 44700, Nepal; ∇ Clean Cooking Alliance, Washington, District of Columbia 20006, United States; ○ Department of Environmental Sciences, Emory University, Atlanta, Georgia 30322, United States; ◆ Institute for Integrated Development Studies (IIDS), Kathmandu 44600, Nepal; ¶ Department of Chemistry, University of Montana, Missoula, Montana 59812, United States; ⋈ Department of Chemical and Biochemical Engineering, University of Iowa, Iowa City, Iowa 52242, United States; $ Department of Environmental Health and Engineering, Johns Hopkins University, Baltimore, Maryland 21218, United States

**Keywords:** ambient aerosol, Nepal, molecular marker, source apportionment, waste burning, secondary organic aerosol

## Abstract

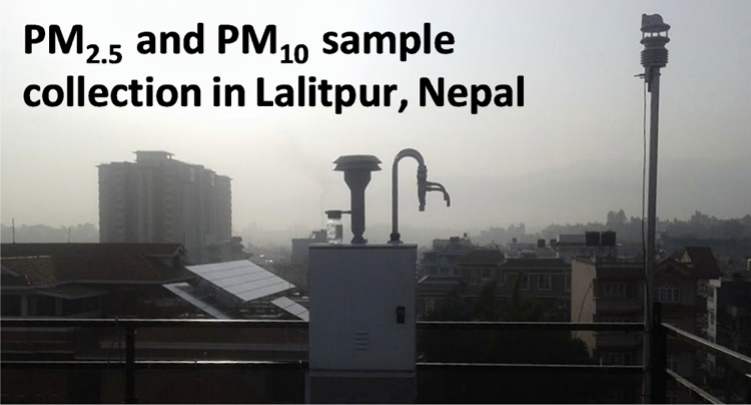

The Kathmandu Valley in Nepal experiences poor air quality, especially in the dry winter season. In this study, we investigated the concentration, chemical composition, and sources of fine and coarse particulate matter (PM_2.5_, PM_10_, and PM_10–2.5_) at three sites within or near the Kathmandu Valley during the winter of 2018 as part of the second Nepal Ambient Monitoring and Source Testing Experiment (NAMaSTE 2). Daily PM_2.5_ concentrations were very high throughout the study period, ranging 72–149 μg m^–3^ at the urban Ratnapark site in Kathmandu, 88–161 μg m^–3^ at the suburban Lalitpur site, and 40–74 μg m^–3^ at rural Dhulikhel on the eastern rim of the Kathmandu Valley. Meanwhile, PM_10_ ranged 194–309, 174–377, and 64–131 μg m^–3^, respectively. At the Ratnapark site, crustal materials from resuspended soil contributed an average of 11% of PM_2.5_ and 34% of PM_10_. PM_2.5_ was largely comprised of organic carbon (OC, 28–30% by mass) and elemental carbon (EC, 10–14% by mass). As determined by chemical mass balance source apportionment modeling, major PM_2.5_ OC sources were garbage burning (15–21%), biomass burning (10–17%), and fossil fuel (14–26%). Secondary organic aerosol (SOA) contributions from aromatic volatile organic compounds (13–23% OC) were larger than those from isoprene (0.3–0.5%), monoterpenes (0.9–1.4%), and sesquiterpenes (3.6–4.4%). Nitro-monoaromatic compounds—of interest due to their light-absorbing properties and toxicity—indicate the presence of biomass burning-derived SOA. Knowledge of primary and secondary PM sources can facilitate air quality management in this region.

## 1 Introduction

Approximately one-third of global premature deaths occurring annually are linked to air pollution and the impact is disproportionately high in South Asia.^1^ The Kathmandu Valley in Nepal experiences severe air pollution due to consistently high levels of particulate matter (PM)^2−6^ and gaseous air pollutants.^7,8^ The Kathmandu Valley is a bowl-shaped basin with surrounding mountains that trap urban air pollutants.^9^ Epidemiological studies demonstrate correlations between high pollution levels in Kathmandu and exacerbations of preexisting respiratory and cardiovascular diseases.^10−12^ Ambient particulate pollution is the third leading cause of lost disability-adjusted life-years (DALY: a measure of overall disease burden, expressed as the number of years lost due to ill health, disability, or early death) in Nepal, with related indoor/household air pollution being second.^13^

PM concentrations in the Kathmandu Valley frequently exceed World Health Organization (WHO) guidelines designed to protect human health when considering annual and daily time scales. Daily average fine particulate matter (PM_2.5_) and PM_10_ concentrations during the premonsoon of 2015 in the Kathmandu Valley were up to 14 times higher than the 2021 WHO 24 h air quality guideline values of 15 and 45 μg m^–3^, respectively.^2^ A 3-year-long study during 2003–2005 showed consistently elevated levels of PM_10_ in the Kathmandu Valley with annual average mass concentrations ranging 166–197 μg m^–3^,^5^ in excess of the 2021 WHO air quality guideline for annual average PM_10_ concentrations of 15 μg m^–3^. A year-long study during 2006 on the outskirts of the Kathmandu Valley demonstrated that PM_2.5_ concentrations were higher during winter months, reaching a maximum in February (∼50 μg m^–3^) and a minimum during the monsoon season (∼10 μg m^–3^).^14^ Shakya et al.^4^ observed a similar pattern of PM_2.5_ concentrations reporting 3.6 times higher concentrations during the dry winter season, on average, compared to the monsoon at an urban residential site located at Lalitpur in the Kathmandu Valley in 2014.

Prior studies in the Kathmandu Valley provided insight into the chemical composition and sources of PM. During the premonsoon (April 2015), PM_2.5_ in the Kathmandu Valley was composed of organic matter (48%), elemental carbon (13%), sulfate (16%), nitrate (4%), ammonium (9%), chloride (2%), potassium (1%), calcium (1%), and magnesium (0.05%).^2^ Using chemical mass balance modeling, major sources of fine particulate organic carbon (PM_2.5_ OC) were identified as garbage burning (18%), biomass burning (17%), vehicle emissions (17%), and naphthalene-derived secondary organic carbon (10%).^2^ Biomass burning was also identified as a major contributor of PM_2.5_ OC at the Godawari site (21%) located at the edge of the Kathmandu Valley, with additional contributions from fossil fuel combustion (7%).^14^ In the winter (December 2012–February 2013), PM_10_ was comprised of organic matter (32%), crustal materials (32%), elemental carbon (8%), and inorganic ions (13%).^6^ Using multivariate receptor modeling, major sources of PM_10_ OC were estimated to be motor vehicles (47%) and biomass and garbage burning (32%).^6^ Total suspended PM samples collected in Kathmandu contained toxic heavy metals, such as Pb, Mn, Cd, Cr, and V that are associated with fossil fuel use and traffic pollution, and crustal elements associated with road dust.^15^ A carbon isotope analysis of PM in the Kathmandu Valley indicated that primary sources accounted for the major fraction (69%) of total suspended particle organic carbon during the winter of 2007–2008.^16^ These studies indicate an important role of combustion, especially motor vehicles, biomass burning, and garbage burning, as sources of PM_2.5_ and dust as a source of PM_10_. Recent chemical mass balance (CMB) source apportionment studies also indicate the need to better understand the secondary organic aerosol (SOA) impacts and precursors.^2,17^

As part of the second Nepal Ambient Monitoring and Source Testing Experiment (NAMaSTE 2), we characterized the chemical composition and the sources of PM at three locations across the Kathmandu Valley in January and February 2018. When combined with combustion source profiles developed in the first NAMaSTE,^18−20^ we gain new insight to (i) the impact of garbage burning on ambient PM; (ii) the precursors to SOA, particularly aromatic VOCs; and (iii) the contributions of trace metals and dust to PM_2.5_ and PM_10_. Our companion paper discusses the composition and sources of nonrefractory PM_1_ measured by aerosol mass spectrometry with high time resolution and colocated measurements of meteorology and gases.^21^

## 2 Methods

### 2.1 Site Descriptions

PM_2.5_ and PM_10_ samples were collected at three air monitoring stations within or near to the Kathmandu Valley, Nepal, during the winter of 2018: Ratnapark (an urban site in Kathmandu), Lalitpur (a suburban site near the south rim of the valley), and Dhulikhel (a rural site located on a ridge at the eastern rim of the valley). Sampling sites were previously established by the International Center for Integrated Mountain Development (ICIMOD) and the Government of Nepal’s Department of Environment (DoEnv). Samplers were positioned to collect representative air samples and were placed atop buildings or other structures when possible. Colocated gas-phase, aerosol, and meteorology measurements are described elsewhere.^21^

Sample collection at Ratnapark (27.706 °N, 85.316 °E; 1300 m a.s.l.), occurred from January 18 to 27, 2018. Ratnapark is a small park surrounded by busy public roads and a bus terminal. The PM sampler was located behind the fence at the north edge of the park ∼1 m above ground level. Sample collection at the Lalitpur site occurred on a rooftop at the ICIMOD headquarters (27.646 °N, 85.324 °E; 1300 m a.s.l.) at ∼10 m above ground level from February 1 to 10, 2018. ICIMOD is surrounded by residential and commercial buildings, with several brick kilns located within 2 km of the sampling site. Sample collection at the Dhulikhel site was conducted atop a water tank 2 m above ground level in a field near the Kathmandu University School of Medical Sciences (27.608 °N, 85.547 °E; 1600 m a.s.l.) from January 7 to 14, 2018. The Dhulikhel site is rural, located on a ridge, just beyond the eastern rim of the Kathmandu Valley ∼25 km southeast of Ratnapark and 8 km east of brick kilns in the Bhaktapur region and away from the town of Dhulikhel. While the Dhulikhel site is often downwind of Kathmandu, it is on a ridge overlooking an adjoining valley in the Kavre District containing three population centers: Dhulikhel, Banepa, and Panauti with a total population of ∼120,000.^22^

### 2.2 PM Sample Collection

Ambient PM_2.5_ and PM_10_ samples were collected using a medium volume sampler (URG-3000 ABC) on to precleaned 47 mm quartz fiber filters (QFF; Tissuquartz, Pall Life Sciences, East Hills, New York) and Teflon filters (Teflo Membrane, 2.0 μm pore size, Pall Life Sciences). Prior to sample collection, QFF were heated to 550 °C for 18 h to remove organic matter. QFF substrates were collected for determination of OC, EC, and organic species, while Teflon substrates were used for the determination of PM mass and metals. The samples were collected during daytime (7:00–18:00) and nighttime (18:30–6:30) intervals (Nepal Standard [local] Time), while the average sunrise and sunset times for this study were 05:55 and 17:35, respectively. The sampled filters were transferred to polystyrene petri dishes lined with precleaned aluminum foil, capped, sealed with Teflon tape, stored frozen in sealed polyethylene bags, and shipped to the University of Iowa for analysis.

### 2.3 PM, OC, and EC Measurements

PM mass concentrations were measured gravimetrically by the difference of pre- and post-sampling masses of Teflon filters using an analytical microbalance (Mettler Toledo XP26) in a controlled environment with respect to temperature (22 ± 0.5 °C) and humidity (31 ± 6%). Filters were conditioned for 48 h prior to mass measurements. Organic carbon (OC) and elemental carbon (EC) were measured on 1.0 cm^2^ subsamples of QFF by a thermal-optical method (OCEC instrument, Model 5L, Sunset Laboratory Inc.) described by Schauer et al.^23^ Uncertainties in OC and EC were calculated following Jayarathne et al.^18^

### 2.4 Extraction and Analysis of Molecular Markers in PM_2.5_

Extraction and analysis of molecular markers in PM_2.5_ samples followed methods detailed elsewhere.^24^ Briefly, organic molecular markers were extracted from QFF with acetonitrile (Fisher Scientific Company, >99.9%). The solvent was evaporated with a rotary evaporator (Heizbad Hei-VAP, Heidolph instruments GmbH & Co.KG, Germany) and a mini evaporator (Reacti-Vap I, Thermo Scientific) under a gentle stream of high-purity nitrogen gas (PRAXAIR Inc.). Subsequently, extracts were filtered (0.2 μm PTFE, Whatman, GE Health Care Life Sciences) and analyzed by gas chromatography (GC, 7890A, Agilent Technologies) mass spectrometry (MS, 5975C, Agilent Technologies). Compounds with hydroxyl and carboxyl functional groups were derivatized with the silylation agent *N*,*O*-bis(trimethylsilyl)trifluoroacetamide with trimethylchlorosilane (BSTFA + TMCS, 99:1, Fluka Analytical 99%) to convert active hydrogen atoms to trimethylsilyl (TMS) groups^25^ before GC-MS analysis. GC temperature programming and the quantification approaches for the organic species are described in Stone et al.^17^ Quantification of organic species was accomplished by internal standard normalized five-point calibration curves of authentic standards or surrogate standards. All of the species concentrations were field blank subtracted and analytical uncertainties of the measurements were propagated from the standard deviation of the field blanks and 20% of the measured concentration to conservatively account for compound recovery from QFF. All samples required dilution to quantify levoglucosan within the linear range of the instrument due to its high concentrations for which an additional 5% uncertainty was added.

### 2.5 Analysis of Trace Metals

Trace metal analysis was performed on PM_2.5_ and PM_10_ Teflon filters collected at the Ratnapark site only. Filters were microwave digested and analyzed by inductively coupled plasma mass spectrometry (ICP-MS) as described in detail elsewhere.^26^ Briefly, filters were placed in modified poly(tetrafluoroethylene) (PTFE-TFM) vessels with 4 mL of concentrated nitric acid (68–70% w/w, Fisher), 1 mL of hydrogen peroxide (30% w/w, Sigma-Aldrich), and 0.375 mL of an internal standard containing yttrium, indium, terbium, and bismuth (PerkinElmer). The vessels were microwaved (Multiwave GO; Anton Paar) with a 30 min ramp to 200 °C followed by a 30 min hold time, following a modified version of EPA Method 3051.^27,28^ The vessels were rinsed 3 times with 50% v/v nitric acid and filtered with acid-rinsed syringe filters (Whatman 0.45 μm PP) into acid-leached 15 mL conical tubes (CentriStar). The samples were reconstituted to 15 mL with 2% nitric acid. The samples were analyzed by ICP-MS (7900 ICP-MS; Agilent). Accuracy and precision of analysis was calculated following the National Environmental Methods Index Standard Method 3125.^29^

### 2.6 Chemical Mass Balance (CMB) Modeling

Source contributions to the PM_2.5_ OC were estimated using the EPA-CMB model (version 8.2) using local and other relevant source profiles and PM_2.5_ measurements as model inputs. Source profiles drawn from the first NAMaSTE^18^ included garbage/plastic waste burning (fire #14), mud stove fueled with hardwood (fire #37―the biomass burning profile in the base-case model result for Dhulikhel), and open biomass burning (fire #39―the biomass burning profile in the base-case model result for Ratnapark and Lalitpur). Other primary source profiles included noncatalyzed gasoline engines,^30^ diesel engines,^31^ and small-scale coal combustion.^32^ The secondary source profiles include isoprene-, monoterpene-, and sesquiterpene-derived SOA;^33^ mono- and diaromatic-derived SOA;^34^ and cresol-derived SOA.^34^

The model sensitivity to the input source profiles was evaluated by substituting a different biomass or garbage burning source profile in the base-case solution. The studied source profiles were developed in NAMaSTE:^18^ a mud stove fueled by hardwood (fire #37), a mud stove fueled by twigs (fire #38), open biomass burning with twigs and cow dung (fire #39), a mud stove fueled by cow dung (fire #40), a mud stove fueled by hardwood and cow dung (fire # 41), and mixed garbage burning (fire #14A and 14B). Model performance was evaluated by the squared correlation coefficient (*R*^2^) that indicates the fit of the source profiles to the ambient data and the weighted sum of the squares of the differences between the measured and modeled concentrations of the fitting species (χ^2^).

### 2.7 Statistical Analysis

Before performing any statistical analysis, data points with values below detection limits were replaced with the limit of detection (LOD)/√2.^35^ The Anderson–Darling normality test was applied. For species that were either normally or log-normally distributed, Pearson’s correlation (*r*) was determined. Two sample *t*-tests were used to compare the means of daytime and nighttime concentrations. Correlation analysis was done in SPSS (version 25), all other statistical tests were performed in Minitab (version 17), and significance was assessed at 95% confidence interval (*p* ≤ 0.05).

## 3 Results and Discussion

### 3.1 PM Concentrations and Composition

**Figure 1:**
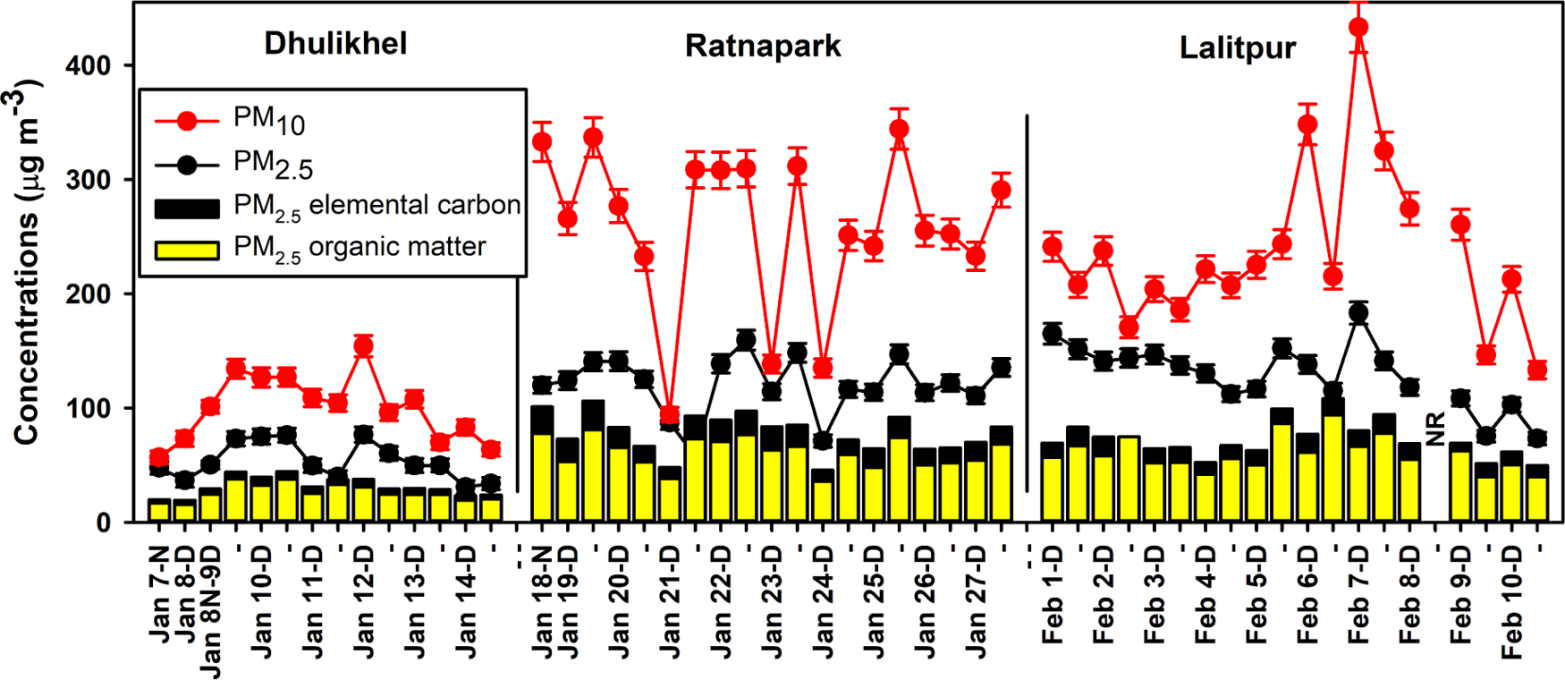
Time series of PM_10_ and PM_2.5_ mass, PM_2.5_ organic matter (1.7 times OC), and elemental carbon at three locations in the Kathmandu Valley in 2018. D denotes daytime samples and N (or dash) represents nighttime samples. Data for the night of February 8 is not reported (NR) due to a sampling error. The difference in PM_10_ and PM_2.5_ represents coarse PM (PM_10–2.5_).

All daily (23 h) PM_2.5_ and PM_10_ concentrations at Ratnapark (January 18–27), Lalitpur (February 1–10), and Dhulikhel (January 7–14) were consistently above the 2021 WHO 24 h guidelines of 15 and 45 μg m^–3^, respectively (Figure 1). The observed PM levels were likewise in excess of the 2005 WHO 24 h guidelines of 25 and 50 μg m^–3^. PM_2.5_ and PM_10_ concentrations at the two in-valley sites (Ratnapark and Lalitpur) were not significantly (*p* = 0.33–0.34) different from each other but were significantly (*p* < 0.001) higher than the more remote Dhulikhel site by a factor of 2.2–2.6 (Table 1). Meanwhile, meteorological measurements, discussed and reported elsewhere, show similar conditions across the three sites, with slightly lower average temperature and relative humidity at Dhulikhel (7.8 °C, 67%, respectively) compared to Ratnapark (10.1 °C, 78%) and Lalitpur (11.2 °C, RH not available).^21^ The average PM_2.5_ concentrations at the in-valley sites (121–130 μg m^–3^) were similar to the average PM_2.5_ concentrations observed in winter to spring of 2014 at six major roadways in the Kathmandu Valley (125 μg m^–3^).^3^

**Table 1:** Summary of PM_10_ Mass; PM_2.5_ Mass; and Select PM Components at Three Sites in the Kathmandu Valley in January–February 2018^a^^,^^b^

	units	Dhulikhel	Ratnapark	Lalitpur
location	degree	27.608 °N, 85.547 °E	27.706 °N, 85.316 °E	27.646 °N, 85.324 °E
altitude	m (a.s.l.)	1600	1300	1300
dates observed in 2018		January 7–14	January 18–27	February 1–10
number of samples		15	19	19
PM_10_ mass	(μg m^–3^)	64.4–131 (102)	194–309 (254)	174–377 (214)
PM_2.5_ mass	(μg m^–3^)	40.4–74.0 (55.2)	71.9–149 (131)	88.3–161 (135)
PM_2.5_ organic carbon	(μg m^–3^)	9.6–20.7 (14.4)	28.0–43.4 (36.1)	26.6–45.7 (36.4)
PM_2.5_ elemental carbon	(μg m^–3^)	3.3–6.6 (4.9)	11.5–22.7 (15.6)	8.5–15.2 (12.8)
OC to EC ratio		2.9–3.4 (3.1)	1.8–2.4 (2.2)	2.4–4.6 (2.8)
1,3,5-triphenylbenzene	(ng m^–3^)	0.43–0.79 (0.60)	2.5–11.9 (3.7)	1.7–6.6 (3.0)
levoglucosan	(ng m^–3^)	666–1248 (912)	1700–2536 (2272)	1069–2125 (1268)
4-nitrocatechol	(ng m^–3^)	19.6–64.0 (52.5)	98.8–362 (202)	68.0–353 (128)
4-methyl-5-nitrocatechol	(ng m^–3^)	5.0–17.4 (13.0)	43.6–122 (81.0)	25.0–145 (47.6)
17α(H)-21β(H)-hopane	(ng m^–3^)	0.16–0.80 (0.35)	1.3–2.1 (1.6)	0.38–1.7 (0.68)
2,3-dihydroxy-4-oxopentanoic acid	(ng m^–3^)	3.7–7.4 (5.8)	4.7–8.7 (6.4)	5.4–9.5 (8.1)

a Daytime and nighttime measurements were averaged to 23 h for comparison with the WHO 24 h PM guidelines. Data are shown as range (and median).

b Average PM_2.5_ and PM_10_ mass concentrations are shown in Table S1.

PM concentrations only had significant day/night variation at the Ratnapark site, where nighttime PM_10_ concentrations were significantly (*p* = 0.018) higher than the daytime concentrations. Meteorological measurements indicated semistagnant winds overnight (typically 0.5 m s^–1^) with a shallow boundary layer of ∼100 m depth compared to higher wind speeds during the day (1.5–2 m s^–1^) with a boundary layer of ∼800 m.^21^ Higher nighttime concentrations of PM in the Kathmandu Valley were observed previously at the Bode site and were explained by a shallower boundary layer, lower wind speed, cooler stagnant air, and favorable partitioning of gases to particles at higher relative humidity at night, especially for compounds with pH-dependent partitioning.^2^

PM_2.5_ to PM_10_ ratios were 0.50, 0.57, and 0.54 at Ratnapark, Lalitpur, and Dhulikhel, respectively, indicating that the fine particles (PM_2.5_) and coarse particles (PM_10–2.5_) were nearly equal in mass concentration. Coarse-mode PM concentrations were relatively high at the two in-valley sites, with PM_10–2.5_ averaging (± standard deviation) 139 ± 54 μg m^–3^ at Ratnapark and 120 ± 60 μg m^–3^ at Lalitpur. A lower average PM_10–2.5_ concentration was documented at Dhulikhel at 47 ± 19 μg m^–3^. The high concentrations of PM_10–2.5_ reveal the importance of coarse particles in contributing to ambient PM in and around the Kathmandu Valley.

Organic carbon (OC) was the most abundant component of PM_2.5_ in all three locations (Figure 1) and contributed on average (± standard deviation) 29 ± 4, 28 ± 7, and 30 ± 7 of PM_2.5_ in Ratnapark, Lalitpur, and Dhulikhel, respectively. Organic matter (OM) includes OC and associated elements (primarily oxygen, hydrogen, and nitrogen) and was estimated by multiplying OC by a factor of 1.7, which was determined by aerosol mass spectrometry (AMS) in a 2015 study in the Kathmandu Valley.^2^ Elemental carbon (EC) accounted for an average (± standard deviation) of 14 ± 3, 10 ± 2, and 10 ± 2% in these three sites, respectively. The average OC to EC ratios were 2.2, 2.8, and 3.1 at Ratnapark, Lalitpur, and Dhulikhel, respectively. OC to EC ratios of 2–4 have been previously observed at urban locations in South Asia^2,36,37^ and are attributed to diesel emissions with OC to EC ratios of 0.64^38^ and low-efficiency biofuel combustion.^39^ The increasing OC to EC ratio at the rural Dhulikhel site reflects a more remote location with smaller vehicle influence^40,41^ and increasing biomass burning and SOA contributions (as discussed in Section 3.3).

### 3.2 PM_2.5_, PM_10_, and PM_10–2.5_ Metal Concentrations at the Ratnapark Site

For metals with WHO guideline values, observed concentrations at the Ratnapark site were well below these thresholds (Table 2). Of the measured metals, 18 of 23 metals (save for Cu, Zn, Mo, Sb, and Pb) correlated very strongly with PM_10_ mass (Table S3), reflecting consistent metal mass fractions in PM_10_, with day-to-day variability attributed to factors that influence the atmospheric loading of PM_10_. Metals such as Cu, Zn, Mo, Sb, and Pb had similar concentrations in PM_2.5_ and PM_10_ (Figure S1), indicating that they derive from combustion sources. Cu strongly correlated with Sb and Pb (Table S3), and these three metals are enhanced in PM_2.5_ emitted from garbage burning.^18^ The concentrations of toxic heavy metals in PM_2.5_ at the Ratnapark site were lower than those observed in other urban locations in Asia, such as Delhi, India;^42,43^ Agra, India;^44^ Lahore, Pakistan;^45^ and Beijing, China^46^ (Table 2). Compared to Lahore, Pakistan, in winter 2007, the lower PM_2.5_ metal concentrations at the Ratnapark site coincided with the lower PM_2.5_ concentrations.

**Table 2:** Daily (23 h) Average Concentrations of PM, Crustal Materials Calculated as Metal Oxides Related to Crustal Origin, and Select Toxic Metals at the Ratnapark Site in Kathmandu from January 18–27, 2018^a^

components	units	Kathmandu, Nepal	Delhi, India	Lahore, Pakistan	Beijing, China	WHO guideline in air
study period		winter 2018	winter 2010	winter 2007	winter 2014	
sample duration	(h)	23	24	24	12	
PM_10_ mass	(μg m^–3^)	194–309 (257)	213	443	200	
PM_10_ crustal material	(μg m^–3^)	52–117 (86.8)		99		
PM_10_ metals						
lead (Pb)	(ng m^–3^)	34.5–67.2 (53.9)		12,000	226	500^b^
manganese (Mn)	(ng m^–3^)	62.0–131 (101)	14	300	120	150^b^
vanadium (V)	(ng m^–3^)	8.3–16.6 (12.8)			11.4	1000^c^
arsenic (As)	(ng m^–3^)	1.7–3.4 (2.7)		22	10.8	1000^d^
chromium (Cr)	(ng m^–3^)	4.5–29.0 (14.3)	370	30	24.9	1000^e^
PM_2.5_ mass	(μg m^–3^)	71.9–149 (121)		295	138	
PM_2.5_ crustal material	(μg m^–3^)	8.8–17.5 (13.5)		13.2		
PM_2.5_ metals						
lead (Pb)	(ng m^–3^)	15.2–64.8 (43.9)		9000	202	
manganese (Mn)	(ng m^–3^)	12.1–27.0 (19.1)		150	70	
vanadium (V)	(ng m^–3^)	1.3–2.8 (2.2)			6.1	
arsenic (As)	(ng m^–3^)	0.90–2.0 (1.7)		10	8.7	
chromium (Cr)	(ng m^–3^)	0.05–16.6 (3.7)		15	12.8	
reference		this study	(42)	(45)	(46)	(48)

a Data are shown as range (and mean) for Kathmandu, Nepal. The metal concentrations at Kathmandu are compared to WHO guideline values and average wintertime concentrations reported for Delhi, India; Lahore, Pakistan; and Beijing, China. Additional metals data is provided in Table S2.

b Applicable to the annual average in air.

c Applicable to the 24 h average in air.

d Associated with a lung cancer risk estimate of 1.5 × 10^–3^ for lifetime exposure.

e WHO guideline applies to hexavalent chromium, which is associated with a lung cancer risk estimate of 4.0 × 10^–2^ for lifetime exposure; however, the chromium concentrations reported here correspond to total chromium. Hexavalent chromium concentrations can be estimated by multiplying the total chromium concentrations by 0.2.^49^

**Figure 2:**
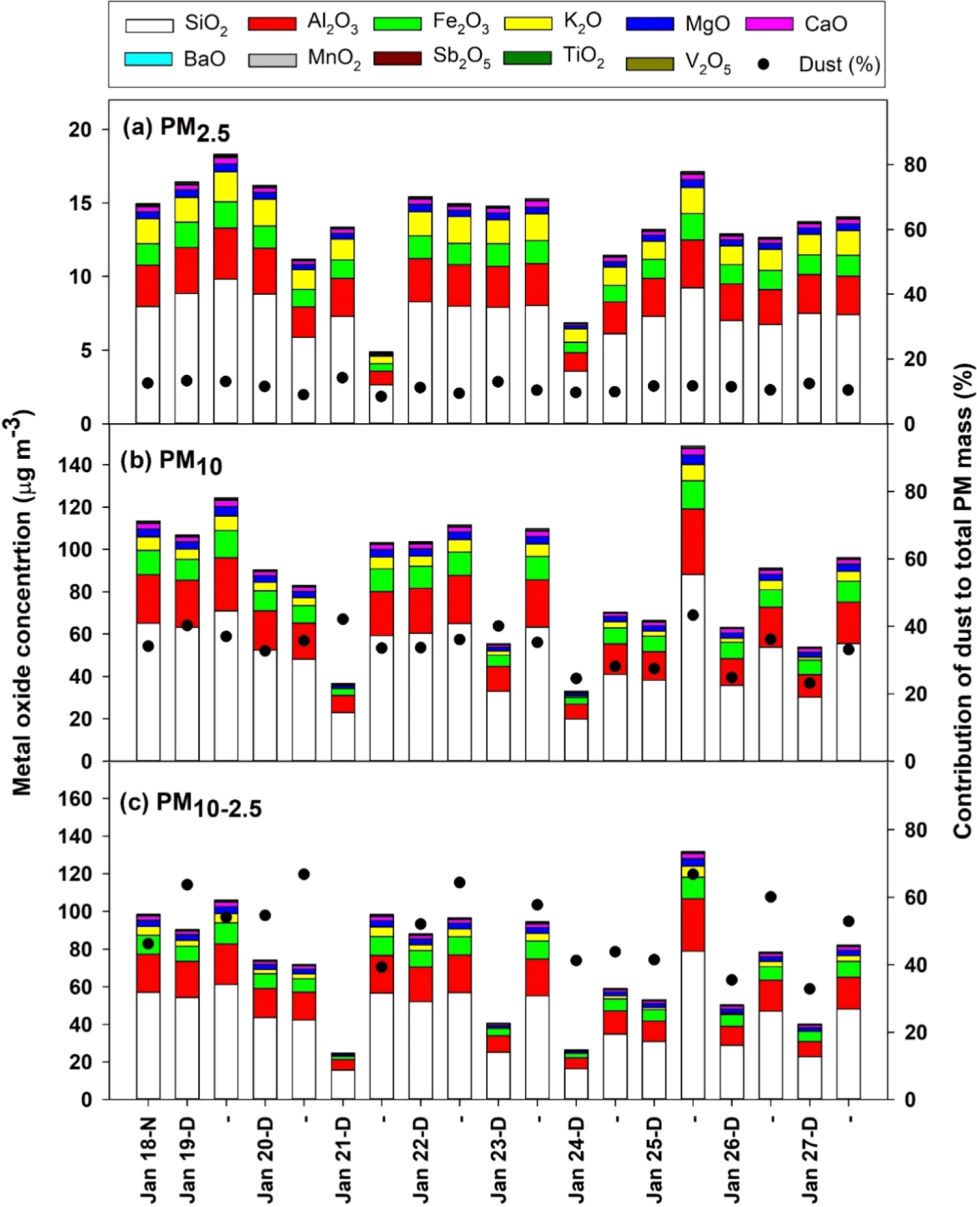
Concentration of dust components and percent contribution of dust to the total PM fraction during January 2018 at the Ratnapark site in Kathmandu, Nepal. Daytime samples are labeled as D and nighttime samples are labeled as N or dash (-).

The contribution of crustal elements to PM_2.5,_ PM_10_, and PM_10–2.5_ mass was estimated using measurements of metals comprising the Earth’s crust: Al, Fe, K, Mg, and Ca. These metals were correlated with one another (Table S3) and were enhanced in PM_10_ relative to PM_2.5_, indicating a significant coarse-mode source (Figure S1). The mass of Si was estimated by the Si–Al ratio (2.5 ± 0.2) of soil in the Nepal Himalaya.^47^ Crustal metals were converted to their most common oxide forms to estimate the dust contribution to PM mass.^45^ On average, crustal material contributed 11.2% of PM_2.5_ mass, 33.7% of PM_10_ mass, and 51.4% of PM_10–2.5_ mass in the Kathmandu downtown area (Figure 2). When averaged to a daily time scale (Table 2), the contribution of dust to PM_10_ exceeded 45 μg m^–3^ on all 10 days, indicating that PM_10_ dust alone exceeds the WHO daily guideline value for PM_10_ during this study. Dust is also an important source of fine particles, contributing a daily average of 13.5 μg m^–3^ of PM_2.5_. Thus, airborne dust mitigation in the Kathmandu Valley provides opportunity for lowering both PM_2.5_ and PM_10_.

### 3.3 Chemical Mass Balance Source Apportionment Modeling of PM_2.5_ Organic Carbon

**Figure 3:**
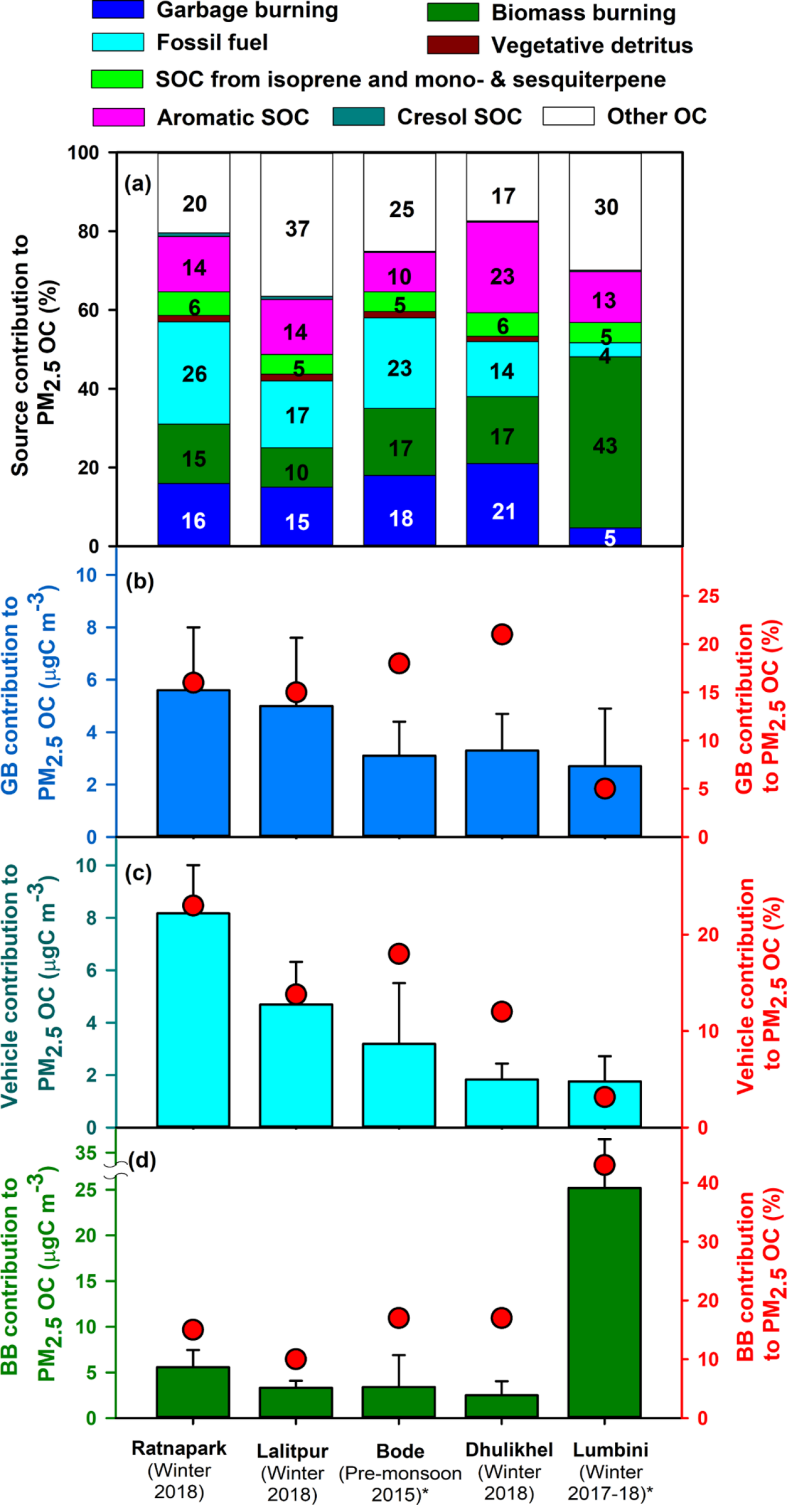
(a) Average percent contributions of source categories to PM_2.5_ OC at the Kathmandu Valley sites during the winter of 2018. The results are compared to prior studies (marked with asterisks): Bode in the Kathmandu Valley during the premonsoon of 2015^2^ and Lumbini in the northern Indo-Gangetic Plain during the winter of 2017–18.^40^ (b) Garbage burning (GB) contributions to PM_2.5_ OC, (c) vehicle (diesel and gasoline) contributions to PM_2.5_ OC, and (d) biomass burning (BB) contributions to PM_2.5_ OC at these sites. Error bars represent one standard deviation. Day-to-day variabilities in source contributions are shown in Figure S3.

Fine particle organic carbon (PM_2.5_ OC) was apportioned to seven primary sources (garbage burning, biomass burning, diesel engines, gasoline engines, coal combustion, natural gas, and vegetative detritus) using chemical mass balance (CMB) modeling. The base-case source apportionment result was obtained by utilizing source profiles (see Section 2.6) and corresponds to our best estimate of source contributions to OC in the Kathmandu Valley. Of the resolved OC, primary sources contributed to 58, 43, and 53% of the total OC at Ratnapark, Lalitpur, and Dhulikhel, respectively. Major primary sources of PM_2.5_ OC across all three sites were garbage burning (15–21%), biomass burning (10–17%), and fossil fuel combustion that included diesel engines, gasoline engines, and coal and natural gas combustion (14–26%) (Figure 3a and Table 3). These major sources agree with our previous source apportionment at Bode in the Kathmandu Valley during the premonsoon of 2015 (Figure 3a).

**Table 3:** Absolute and Relative Contributions of Primary and Secondary Sources to PM_2.5_ OC (± Standard Error) during the Winter of 2018 in the Kathmandu Valley

	Dhulikhel (*n* = 15)		Ratnapark (*n* = 19)		Lalitpur (*n* = 19)	
source category	(μg OC m^–3^)	PM_2.5_ OC (%)	(μg OC m^–3^)	PM_2.5_ OC (%)	(μg OC m^–3^)	PM_2.5_ OC (%)
garbage/plastic burning	3.3 (0.4)	21.2 (1.3)	5.6 (0.6)	15.6 (1.3)	5.0 (0.6)	15.2 (2.0)
biomass burning	2.5 (0.4)	17.0 (2.5)	5.6 (0.4)	15.4 (0.8)	3.3 (0.2)	9.6 (0.5)
diesel engines	1.7 (0.1)	10.9 (0.5)	6.0 (0.4)	16.8 (0.6)	4.6 (0.2)	13.5 (0.8)
gasoline engines	0.22 (0.05)	1.3 (0.3)	2.2 (0.2)	6.2 (0.4)	0.52 (0.15)	1.3 (0.3)
coal combustion	0.13 (0.02)	0.81 (0.13)	0.30 (0.03)	0.85 (0.08)	0.29 (0.05)	0.77 (0.12)
natural gas	0.17 (0.03)	1.1 (0.2)	0.65 (0.06)	1.8 (0.1)	0.70 (0.07)	2.0 (0.1)
vegetative detritus	0.19 (0.01)	1.3 (0.1)	0.56 (0.07)	1.6 (0.2)	0.65 (0.10)	1.7 (0.2)
isoprene SOC	0.066 (0.003)	0.44 (0.02)	0.10 (0.01)	0.29 (0.01)	0.17 (0.01)	0.50 (0.01)
monoterpene SOC	0.21 (0.01)	1.4 (0.05)	0.38 (0.05)	1.0 (0.1)	0.30 (0.03)	0.86 (0.10)
sesquiterpene SOC	0.65 (0.04)	4.4 (0.3)	1.5 (0.1)	4.3 (0.2)	1.3 (0.1)	3.6 (0.2)
monoaromatic SOC	2.2 (0.1)	14.4 (0.7)	2.5 (0.2)	7.2 (0.4)	3.0 (0.2)	8.4 (0.2)
diaromatic SOC	1.2 (0.1)	8.2 (0.5)	2.0 (0.2)	5.6 (0.4)	1.7 (0.2)	5.0 (0.5)
cresol SOC	0.048 (0.006)	0.30 (0.03)	0.36 (0.05)	1.0 (0.1)	0.29 (0.05)	0.77 (0.09)
other OC	2.9 (0.7)	17.8 (3.1)	8.2 (0.9)	22.5 (1.9)	14.0 (1.8)	37.8 (3.2)

In general, there was a decreasing trend in both absolute and percent contributions of garbage burning (Figure 3b) and vehicle emissions (Figure 3c) to PM_2.5_ OC from urban to rural sites (from Ratnapark, Lalitpur, Bode, Dhulikhel, and Lumbini). This observation agreed with the realistic expectations that the combustion of garbage and vehicle emissions are more abundant in urban areas with higher population density and traffic. Although Dhulikhel and Bode had lower absolute impacts from garbage burning, they had a higher relative contribution from this source compared to Ratnapark and Lalitpur sites (Figure 3b). In contrast, there was a consistent increasing trend in the percent contribution of biomass burning to PM_2.5_ OC from urban to rural sites in Nepal (Figure 3d), indicating that in rural areas of Nepal, this source was relatively more abundant compared to urban areas. This is further supported by the agroresidue burning observed at Lumbini during the winter period of 2017–2018 described in our companion paper.^40^

PM_2.5_ OC was also apportioned to six types of secondary organic carbon (SOC) based on the precursors (isoprene SOC, monoterpene SOC, sesquiterpene SOC, monoaromatic SOC, diaromatic SOC, and cresol SOC) using the SOC tracer-based method described in Kleindienst et al.^33^ These assigned SOC sources contributed significantly to total PM_2.5_ OC at Ratnapark (19%), Lalitpur (19%), and Dhulikhel (29%). SOC from mono- and diaromatic VOCs was the largest assigned SOC precursor class at all three sites (Figure 3a), with the largest relative contribution at Dhulikhel (23 ± 4%). SOC from isoprene, monoterpene, and sesquiterpene precursors was much lower than SOC from aromatic precursors. Isoprene in the Kathmandu Valley during winter was mainly attributed to vehicle emissions and biomass burning by prior studies^50,51^ and not biogenic emissions. Among the isoprene tracers, the sum of 2-methylthreitol and 2-methylerythritol, formed under lower NO*_x_* conditions,^52^ was higher at the rural Dhulikhel site, whereas 2-methylglyceric acid, formed under higher NO*_x_* conditions, was more abundant at the urban in-valley sites (Figure S2). The predominance of 2-methylglyceric acid has been previously observed in the Kathmandu Valley^17^ and other urban sites^53^ and may reflect a relatively large influence of NO*_x_* on isoprene SOA formation at the in-valley sites, where the NO*_x_* level of ∼79 ppbv was much higher than rural Dhulikhel at ∼9 ppbv.^21^

OC not apportioned to the above primary or secondary sources is referred to as “other sources” in Figure 3a and contributed significantly at Ratnapark (22%), Lalitpur (38%), and Dhulikhel (18%). These other sources may be primary including mixed industrial emissions, cooking with other types of solid fuels (e.g., bio-briquettes), OC associated with airborne dust, and any other sources for which profiles were not available (i.e., local industries, local food cooking). Other sources can include those for which profiles exhibited colinearity and could not be included in the model (e.g., brick kilns were excluded because they were colinear with biomass burning). Unapportioned OC may also result from variability in the selected profiles and actual source emissions impacting each site, which is further examined through sensitivity tests of biomass and garbage burning source profiles (discussed in Sections 3.4 and 3.5). In addition, unapportioned OC can result from a source not represented in the CMB model, which could be primary (e.g., brick kilns) or secondary (i.e., SOC from other VOCs emitted from biomass burning).^54^ Unapportioned OC was highest for the Lalitpur location, which suggests that additional sources such as brick kilns^21^ are present or that the selected profiles were not representative of the emissions impacting the sampling site. Building upon prior source apportionment studies in the Kathmandu Valley,^2,14^ this work introduces additional source profiles (i.e., natural gas, monoaromatic SOC, and cresol SOC) and provided more complete mass closure on PM_2.5_ OC.

### 3.4 Evaluation of the Garbage Burning Contribution in the Kathmandu Valley and Understanding the Spatial Variability

**Figure 4:**
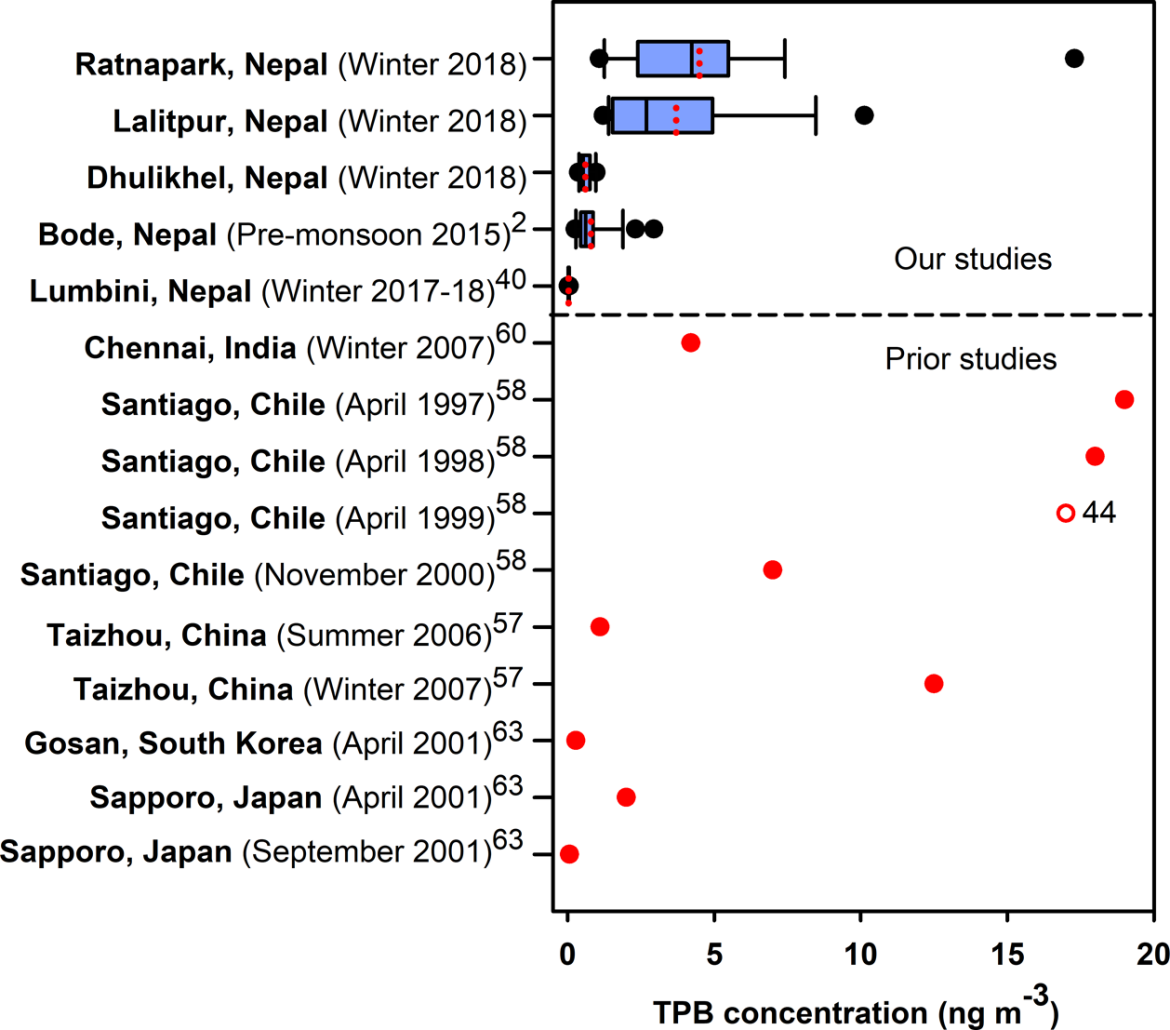
Shown in the box plots are concentrations of TPB at Ratnapark, Lalitpur, and Dhulikhel in the Kathmandu Valley during January–February 2018 (this study), Bode in the Kathmandu Valley during the premonsoon of 2015, and Lumbini in the northern Indo-Gangetic Plains during December 2017–January 18. The box plots show the 25th and 75th percentiles (box), range (bars), median (black vertical line), mean (red dotted line), and outliers (black dots). Mean concentrations (filled red dots) and one off-scale value (open red circle) are shown for Islam et al.,^2^ Islam et al.,^40^ Fu et al.,^60^ Simoneit et al.,^58^ Gu et al.,^57^ and Simoneit et al.^63^

Open garbage burning is a common way to dispose of waste materials in Nepal and other South Asian countries,^55^ and is a substantial contributor to gaseous and particulate air pollutants.^56^ 1,3,5-Triphenylbenzene (TPB) has been used as a molecular marker for garbage burning^2,40,57^ because it is uniquely produced by the combustion of plastic^18,58^ that makes up ∼10% of global garbage.^59^ TPB was consistently detected at substantial levels at all three sampling locations in or near to the Kathmandu Valley during the winter of 2018 (Table 1). TPB concentrations were higher at the in-valley sites (Ratnapark, Lalitpur, and Bode) compared to rural Dhulikhel at the eastern valley rim and rural Lumbini (Figure 4). Relatively high TPB concentrations have been observed in urban sites in Chennai, India;^60^ Santiago, Chile;^58^ and downwind of an e-waste dismantling area at Taizhou, China,^57^ reflecting higher population density and quantity of garbage burned.

Antimony (Sb) is also emitted from burning plastic and plastic-containing materials such as textiles^18,56,61^ and has been used as an elemental tracer for garbage burning.^56,62^ On average, 91% of Sb at the Ratnapark site was in PM_2.5_, while the remainder was in coarse particles (PM_10–2.5_), indicating that the majority of Sb originated from combustion. To the best of our knowledge, this is the first report of colocated measurements of TPB and Sb in ambient PM. The Sb-to-TPB ratios in ambient PM_2.5_ are within the range of those observed in local source emissions studied in NAMaSTE 1 field campaign (Figure S4a). Plastic waste had the lowest Sb-to-TPB ratios, while foil wrappers had the highest, and mixed-waste source emissions and ambient PM_2.5_ measurements were in between. The Sb-to-TPB ratios for ambient PM_2.5_ were scattered, likely due to differing garbage composition and/or burning conditions. The log-transformed concentrations of Sb and TPB in ambient PM_2.5_ were moderately correlated (*r* = 0.50, *p* = 0.028, Figure S4b), consistent with their cooccurrence in garbage burning emissions.

Based on CMB modeling, garbage burning is one of the major contributors of PM_2.5_ OC in the Kathmandu Valley contributing on average 5.0–5.6 μgC m^–3^ at the in-valley sites and 3.3 μgC m^–3^ at Dhulikhel (Table 3). Day-to-day variability was substantial and the daily estimated contribution of this source to the PM_2.5_ OC reached up to 9.1 μgC m^–3^ (28%) at the in-valley sites and 5.8 μgC m^–3^ at Dhulikhel (30%). While the average percent contribution of garbage burning to PM_2.5_ at the in-valley sites was similar to that during the premonsoon of 2015 in the Valley (Figure 3b),^2^ the absolute contribution of garbage burning to OC was approximately double in 2018, consistent with higher PM_2.5_ concentrations. The present study supports the importance of garbage burning to the air quality in this region,^40,55,60,64,65^ and provides a quantitative estimation of its impact on air quality in the Kathmandu Valley. The large and consistent contribution of this source to the air quality suggests that this source needs to be considered in future air quality studies in this region and also in many other regions where open garbage burning is common.

The sensitivity of the CMB source apportionment results to the input garbage burning source profiles was examined using two garbage burning source profiles while keeping other profiles constant, following prior studies.^2,37,66^ Two garbage burning profiles were tested from a single fire of mixed-waste burning (NAMaSTE fire nos. 14A and 14B).^18^ The mixed waste included food waste, paper, plastic bags, cloth, diapers, and rubber shoes. Profile B was used as the base case that corresponded to a mixture of flaming and smoldering combustion, while profile A was used in the sensitivity test and corresponds to more smoldering conditions.^18^ Switching from profile B to A increased the amount of PM_2.5_ OC apportioned to garbage burning by factors of 1.1, 1.5, and 1.9 for Dhulikhel, Ratnapark, and Lalitpur, respectively (Figure S5), indicating that the model was reasonably stable to the garbage burning profile for Dhulikhel but moderately sensitive for in-Valley sites. The sensitivity result indicated that this source may contribute to more OC than was estimated in the base-case scenario and may account for some unapportioned OC.

The CMB source contribution for garbage burning was influenced by a combination of tracers including TPB, C_30_ and C_32_*n*-alkanes, and benzo(ghi)perylene. The influence of C_30_ and C_32_ alkanes on OC apportionment for garbage burning was due to the even carbon preference for *n*-alkanes in plastic burning emissions, which results from the production of low-molecular-weight oligomers during polymerization of ethylene.^58^ The influence of benzo(ghi)perylene was consistent with higher abundance of this species in garbage burning emissions compared to emissions from other sources in Nepal.^18^ Our CMB source apportionment of garbage burning that included all of these molecular markers better constrained the garbage burning source contribution than a single tracer and this approach can be recommended for future studies.

### 3.5 Biomass Burning Contributions to PM_2.5_ OC and Insight to Biomass Burning Impacts on SOA

**Figure 5:**
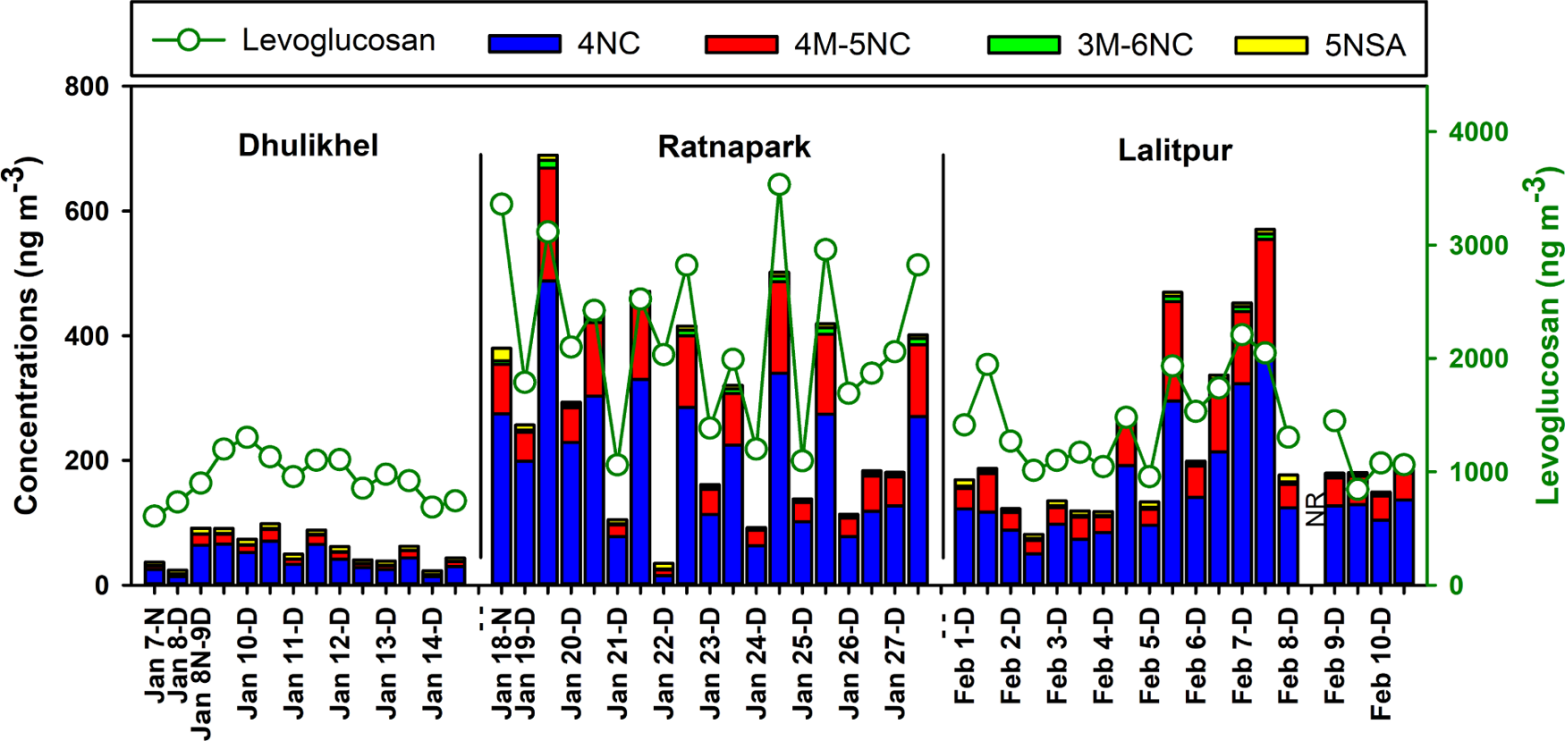
Time series plot of levoglucosan and nitroaromatic compounds (NACs): 4-nitrocatechol (4NC), 4-methyl-5-nitrocatechol (4M-5NC), 3-methyl-6-nitrocatechol (3M-6NC), and 5-nitrosalicylic acid (5NSA). Data on February 8 night is not reported (NR) as the sampling time was unknown for that sample.

Biomass burning in Nepal includes biofuel use, especially for cooking, as well as agricultural waste burning, with the former expected to dominate in the Kathmandu Valley during wintertime. During dry years, there are also significant contributions from forest fires. Levoglucosan, a cellulose pyrolysis product and a well-established molecular marker of biomass combustion,^67^ is used to track the biomass burning impact on atmospheric particulate matter in the Kathmandu Valley. Levoglucosan concentrations were very high at all three sites during the winter of 2018 (Figure 5). The highest concentration of levoglucosan was observed at the Ratnapark site, which had a median levoglucosan concentration approximately 2 and 2.5 times higher than the suburban Lalitpur and rural Dhulikhel sites, respectively (Table 1). This was most likely due to local sources in the Kathmandu urban core including some food vendors who used wood fires near this site. The average levoglucosan concentration at the Ratnapark site (2201 ng m^–3^) was also ∼10 times higher than the average levoglucosan concentration at Godawari located on the outskirts of the Kathmandu Valley during the winter of 2006.^14^

For the base-case source apportionment modeling, the one-pot traditional mud cooking stove fueled with hardwood (fire no. 37)^18^ was used as the biomass burning profile for the Dhulikhel site because of the predominance of hardwood as fuel in this location.^68^ At the Ratnapark and Lalitpur sites, the open fire with twigs and dung (fire no. 39)^18^ was used as the base-case biomass burning profile because (i) this profile was reported as the best fit of available biomass burning profiles for the Kathmandu Valley in a prior study,^2^ (ii) wood was reported to be the most common solid biomass fuel inside the Valley,^69^ and (iii) a substantial concentration of stigmastanol (a dung tracer) was observed at Ratnapark and Lalitpur sites (Table S4). Additionally, stigmastanol-to-OC ratios at the Ratnapark and Lalitpur sites were 1.4–1.5 times higher compared to the Dhulikhel site. Because dung fires are not common in Kathmandu Valley, the observed stigmastanol may come from regional emissions transported to these sites or from a noncombustion process. Based on the CMB source apportionment modeling, biomass burning contributed an average of 3.3–5.6 μgC m^–3^ at the in-valley sites and 2.5 μgC m^–3^ at Dhulikhel (Table 3). Similar to the trend of levoglucosan concentrations, the biomass burning contribution to PM_2.5_ OC at the Ratnapark site was approximately 1.7 and 2.2 times higher than the suburban Lalitpur and rural Dhulikhel sites, respectively. Day-to-day variability showed that biomass burning can contribute up to 8.6 μgC m^–3^ (25%) to the PM_2.5_ OC inside the valley as observed at the Ratnapark site and 5.5 μgC m^–3^ (30%) at Dhulikhel on a daily basis. The average percent contribution of this source to PM_2.5_ OC at the Ratnapark site (15%) was approximately the same as the Bode site (17%) in the Kathmandu Valley during the premonsoon of 2015,^2^ while the absolute concentration was approximately double in this study (Figure 3d).

The sensitivity of the CMB source apportionment model to the input biomass burning source profiles was examined using three additional biomass burning source profiles while keeping other source profiles constant. The examined biomass burning profiles were chosen from NAMaSTE:^18^ open fire with twigs and dung [fire no. 39], one-pot traditional mud cooking stove fueled with hardwood [fire no. 37], twigs [fire no. 38], and hardwood and dung [fire no. 41]. For Dhulikhel, switching the biomass profile from fire no. 37 to other profiles decreased the amount of PM_2.5_ OC apportioned to biomass burning by factors of 0.71–0.97 corresponding to 3–29% reduction (Figure S7). Meanwhile, for Ratnapark and Lalitpur, switching the biomass profile from fire no. 39 to other profiles decreased the amount of PM_2.5_ OC apportioned to biomass burning by factors of 0.10–0.63 corresponding to 37–90% reduction. In an additional test, the model was very sensitive when using the source profile for the one-pot traditional mud cooking stove fueled with dung (fire no. 40),^18^ which increased PM_2.5_ OC apportioned to this source by factors of 2.3–5.0 over the three locations, likely because biomass burning in the Kathmandu Valley was not well-represented by this source profile. These results indicated that source apportionment to biomass burning was moderately sensitive to the selected biomass burning profiles for Dhulikhel and was very sensitive at Ratnapark and Lalitpur. For these in-valley sites, the base-case model is an upper estimate of biomass burning and has a larger relative uncertainty. This uncertainty arises from the diversity of biofuel use in the valley, including cooking fires, open fires, and brick kilns, as well as the combustion of cellulose-based materials in garbage.

**Figure 6:**
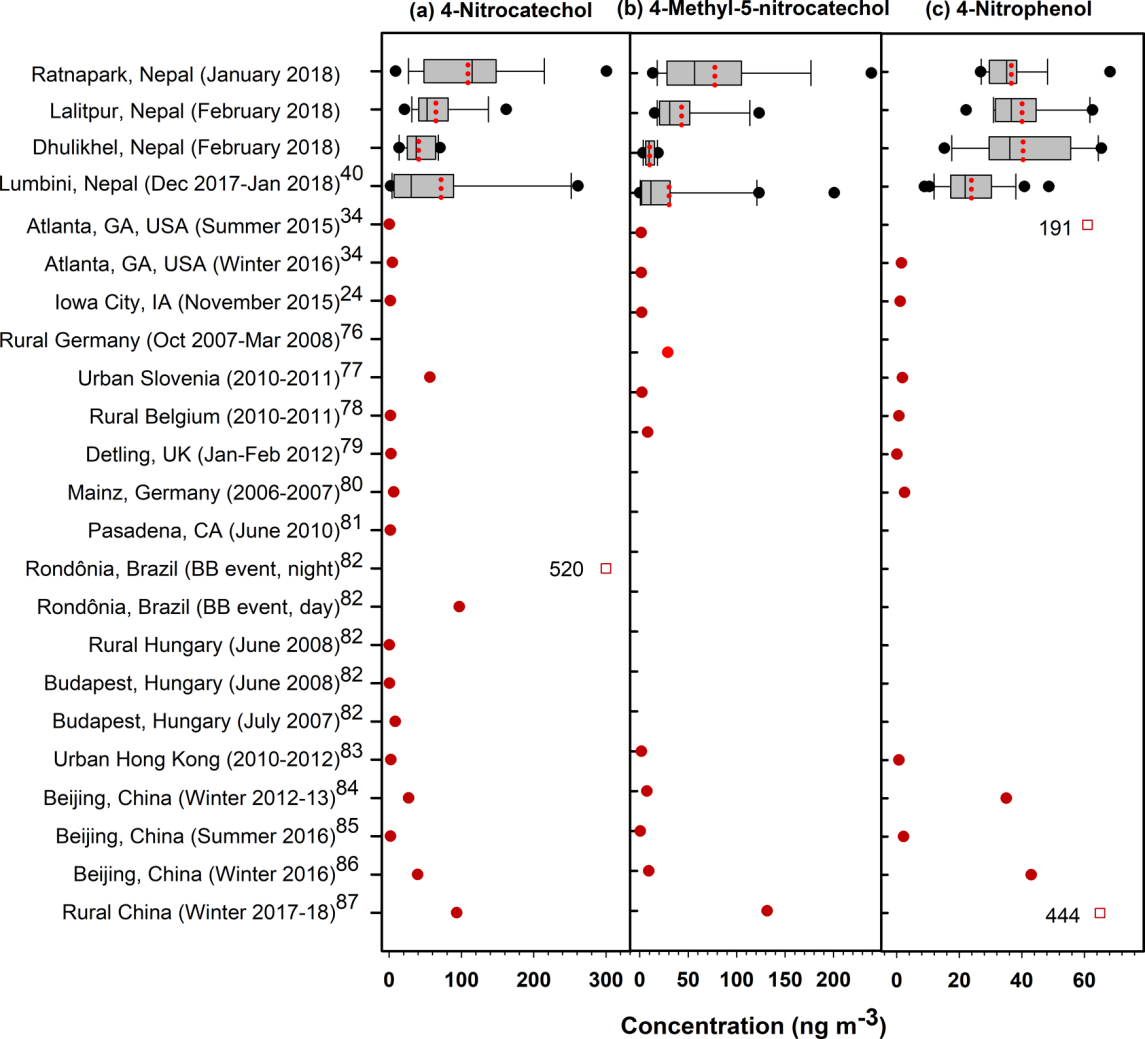
Box plot of NACs in the Kathmandu Valley sites and comparison with prior studies. The Kathmandu Valley and Lumbini plots show the 25th and 75th percentiles (box), range (bars), median (black vertical line), mean (red dotted line), and outliers (black dots). Mean concentrations (filled red dots) and two off-scale values (open red squares) are shown for Islam et al.,^40^ Al-Naiema and Stone,^34^ Al-Naiema and Stone,^24^ Iinuma et al.,^76^ Kitanovski et al.,^77^ Kahnt et al.,^78^ Mohr et al.,^79^ Zhang et al.,^80^ Zhang et al.,^81^ Claeys et al.,^82^ Chow et al.,^83^ Ma et al.,^84^ Wang et al.,^85^ Li et al.,^86^ and Salvador et al.^87^

Insight to SOA derived from biomass burning was gained through four nitroaromatic compounds (NACs: 4-nitrocatechol, 4-methyl-5-nitrocatechol, 3-methyl-6-nitrocatechol, and 5-nitrosalicylic acid) that are atmospheric oxidation products of phenolic compounds^34,70^ emitted from lignin pyrolysis during biomass burning.^71−73^ Like levoglucosan, the NAC concentrations at the Ratnapark site were comparable with the concentrations at Lalitpur, but 5–8 times higher than at Dhulikhel, except for 5-nitrosalycylic acid that was comparable at all sites (Figure 5). The summed concentrations of these four NACs reached up to 690 ng m^–3^ at the Ratnapark site and 98 ng m^–3^ at Dhulikhel (Table S4). The average concentrations of 4-nitrocatechol and 4-methyl-5-nitrocatechol at the Ratnapark site were among the highest reported concentrations, following Rondônia, Brazil, during a biomass burning event in the Amazon rainforest and rural China (Figure 6). These four NACs showed moderate to high correlations among themselves (*r* = 0.68–0.99; *p* < 0.001; Table S5), supporting their formation from a common source. Their correlation with levoglucosan (*r* = 0.55–0.82; *p* < 0.001; Table S5) is consistent with their origin being biomass burning. According to a chamber experiment, NACs derived from *m*-cresol were major components of brown carbon contributing ∼50% to the total light absorption coefficient at 365 nm.^74^ Thus, these nitrocatechols are expected to contribute to the brown carbon that has been previously demonstrated to be abundant in the Kathmandu Valley.^19,75^ Colocated measurements of brown carbon (reported elsewhere^21^) were moderately correlated with NACs at the Ratnapark site, but not at Dhulikhel (Table S5), which may reflect higher NO*_x_* levels in the urban Ratnapark site compared to Dhulikhel.

Using the NACs in the SOA tracer-based method,^34^ the contribution of cresol-derived SOC to PM_2.5_ OC was estimated to be on average 0.29–0.36 μgC m^–3^ (0.8–1%) at the in-valley sites and 0.05 μgC m^–3^ (0.3%) at Dhulikhel. The SOC (μgC m^–3^) contributed by the cresol SOA at the in-valley sites were approximately double the levels observed in Lumbini during the winter of 2017–18.^40^ The absolute and relative impact of primary biomass burning emissions was larger in Lumbini compared to the Kathmandu Valley sites (Figure 3), such that the higher NACs in Kathmandu suggest that local urban influences on SOA result from higher NO*_x_* and oxidant levels in the valley. Importantly, NACs account for only the fraction of SOC from biomass burning that is associated with cresol derivatization and they do not represent all biomass burning SOC. A more complete assessment of biomass burning impacts on SOC would require further expansion of the tracer-based method (including more precursors) or use of other source apportionment approaches, such as multivariate modeling.

### 3.6 Vehicle Fleet Contribution to PM_2.5_ OC

Gasoline and diesel are used in more than 1 million vehicles and 0.25 million generators in the Kathmandu Valley.^88^ Three hopanes (17α(H)-21β(H)-hopane, 17β(H)-21α(H)-30-norhopane, 17α(H)-22,29,30-trisnorhopane) were used to track fossil fuel contributions to the PM_2.5_ OC. The highest concentrations of hopanes were observed at the Ratnapark site with combined concentrations of the three hopanes ranging from 2.8 to 6.4 ng m^–3^ and averaging 4.4 ± 1.1 ng m^–3^. The summed concentrations of these three hopanes at the Ratnapark site were 2.3 and 5.8 times higher than the average summed concentrations observed at Lalitpur and Dhulikhel, respectively, indicating that the fossil fuel impact on ambient PM_2.5_ was much higher at the urban Ratnapark site (located near busy roadways and a bus depot) compared to suburban Lalitpur and rural Dhulikhel.

Together, emissions from gasoline and diesel engines were the largest contributor of PM_2.5_ OC at the Ratnapark site (Figure 3a). Referred to as the vehicle contribution, this source category was estimated to contribute 8.2 μgC m^–3^ (23%) of PM_2.5_ OC at the Ratnapark site, 4.7 μgC m^–3^ (14%) at Lalitpur, and 1.8 μgC m^–3^ (12%) at Dhulikhel (Figure 3c). Gasoline and diesel engines were also the largest contributor to PM_2.5_ OC at the Bode site (23%) in the Kathmandu Valley during the premonsoon of 2015;^2^ however, they were a minor primary source at rural Lumbini during the winter of 2017–18 (Figure 3a).^40^ The large contribution of this source to PM_2.5_ OC in the Kathmandu Valley signifies that implementation of more stringent emissions standards for vehicles could significantly reduce PM_2.5_ OC levels in the Kathmandu Valley.

### 3.7 Natural Gas Contribution to PM_2.5_ OC

In Nepal, natural gas is mainly used in the form of liquified petroleum gas (LPG), which is gaining popularity as household cooking fuel in the Kathmandu Valley.^89,90^ The contribution of natural gas to PM_2.5_ OC was much smaller (on average 1.1–2.0%) than biomass burning (on average 10–17%) in all three locations in the Kathmandu Valley (Table 3). This is most likely due to natural gas having over 40 times smaller PM_2.5_ emission factor (9.5 mg MJ^–1^) than wood (408 mg MJ^–1^)^68^ and more widespread use of solid fuels over natural gas across Nepal.^89^ A comparison between the source apportionment results with and without natural gas showed that exclusion of the natural gas profile in the CMB modeling increased the OC apportioned to garbage burning by factors of 1.3–1.7 and the OC apportioned to coal combustion by factors of 1.2–2.1 and decreased the unapportioned OC by factors of 0.63–0.83 (Figure S9). These results indicated that, in the absence of the natural gas profile, the CMB model assigned too much OC to garbage burning and coal combustion. The source apportionment results with natural gas included were considered the best estimate and reported as the base case in this study because natural gas is a fuel in the Kathmandu Valley. In addition to that, inclusion of this source yielded better CMB model fit parameters (*R*^2^ and χ^2^), averaging 0.82 and 5.18, respectively, when natural gas was included, compared to 0.79 and 5.31, respectively, when it was excluded. An increase in the *R*^2^ value and a decrease in the χ^2^ value were indicative of a better fit between measured and calculated OC.^91^

### 3.8 Aromatic SOA Tracers and Their Contributions to PM_2.5_ OC

SOA from aromatic volatile organic compounds (VOCs) was examined using 2,3-dihydroxy-4-pentanoic acid (DHOPA), phthalic acid, and 4-methyl-phthalic acid. Herein, we present the first measurements in the Kathmandu Valley of DHOPA (also known as T3), which is an oxidation product of toluene,^92^ and other monoaromatic hydrocarbons.^34^ DHOPA was consistently detected at all three sites in the study. The highest 24 h median concentration of DHOPA was observed at Lalitpur (8.1 ng m^–3^), which was 1.3 and 1.4 times higher than the 24 h median concentrations observed at Ratnapark and Dhulikhel, respectively (Table 1). The average concentration (±1 standard deviation) of DHOPA at the Ratnapark site (6.6 ± 1.6 ng m^–3^) was 2.7 times lower than Lumbini located in the northern IGP during the winter of 2017–18. The higher DHOPA concentration in Lumbini may result from the relatively larger biomass burning impact on PM_2.5_ in the IGP, higher toluene concentrations from urban areas upwind, and/or a greater extent of SOA formation compared to the Kathmandu Valley. The latter most likely occurred due to predominant formation of DHOPA from aromatic VOCs under lower NO*_x_* levels at Lumbini^40^ compared to the Kathmandu Valley^21^ as observed in several chamber experiments.^33,34,93,94^

Ambient concentrations of DHOPA were used to estimate SOC contributions of the precursor VOCs to PM_2.5_ OC.^24,33,34^ SOC derived from aromatic VOCs was estimated using the revised SOA tracer-based method described by Al-Naiema et al.^34^ in which the traditional *f*_SOC_ (tracer to SOC ratio) was replaced with weighted-average *f*_SOC_ (*f*_SOC_’) to include other monoaromatic VOC (i.e., benzene, xylenes) in addition to toluene in determining *f*_SOC_. Among the investigated SOC tracers, the sum of the monoaromatic VOCs was determined to be the largest SOC contributor at all three sites (Table 3). Following the spatial trends in DHOPA concentrations, the largest contribution of monoaromatic SOC was observed at Lalitpur (3.0 μgC m^–3^) followed by Ratnapark (2.5 μgC m^–3^) and then Dhulikhel (2.2 μgC m^–3^).

Phthalic acid is an SOA tracer for diaromatic VOCs, such as naphthalene and 1-methylnaphthalene.^34,95^ Because phthalic acid can also form from o-xylene, the ambient concentration of phthalic acid was corrected for the *o*-xylene contribution prior to estimating diaromatic SOC. The portions of ambient phthalic acid estimated to form from *o*-xylene were 34, 28, and 34% at Dhulikhel, Ratnapark, and Lalitpur, respectively (for details, see Section S1). SOC derived from diaromatic VOCs was the second largest identified source of SOC (Table 3), with SOC contributions of 2.0 μgC m^–3^ at Ratnapark, 1.7 μgC m^–3^ at Lalitpur, and 1.2 μgC m^–3^ at Dhulikhel. Together, SOC from monoaromatic and diaromatic precursors accounted for an average of 14–23% of the total PM_2.5_ OC in these locations (Figure 3a). In a prior study during 2012–2013, over 60% of the toluene, C_8_, and C_9_ aromatic VOCs was attributed to traffic-related emissions and 20% was apportioned to residential biofuel use, waste burning, and biomass cofired brick kilns.^51^ Thus, the reduction of emissions from these sources is not only expected to reduce the contribution to primary PM but also to reduce SOA.

## 4 Environmental Implications

Air quality in the Kathmandu Valley was very poor in January–February 2018 as demonstrated by the consistent exceedances of PM_2.5_ and PM_10_ concentrations above the WHO guidelines, with the highest PM levels observed at the two in-valley, urban sites, and lower levels observed at the rural Dhulikhel site. At the Ratnapark site in Kathmandu, resuspended dust contributed substantially to PM, accounting for an average of 51% of coarse particle mass (PM_10–2.5_) and a median of 34% of the PM_10_ mass concentration. Organic carbon (OC) was the largest component of PM_2.5_ in all three locations, with garbage burning, biomass burning, and vehicles being major primary OC sources. The total SOA contribution to PM_2.5_ OC that could be assigned to specific precursor compounds was minor compared to total OC that could be assigned to primary sources. Aromatic VOCs contributed an amount of secondary OC similar in magnitude to the OC emitted by major individual primary sources. Impacts of biomass burning SOA were observed through NACs derived from cresol oxidation, but the full suite of biomass burning precursors has not yet been evaluated. While the majority of PM_2.5_ OC was assigned to primary or secondary source categories, a significant portion of OC remained unapportioned (ranging 17–37% at the three sites), suggesting that some additional SOA and/or source characterization (e.g., traffic in Kathmandu, local industries) are needed to fully represent and reconstruct ambient PM_2.5_ OC sources in Kathmandu.

The Kathmandu Valley Air Quality Management Action Plan, passed by the Nepali government in 2020, is designed to improve air quality in the region. This plan outlines several measures that are designed to reduce ambient air pollution, including PM. For example, to reduce transportation emissions, the plan calls for implementing the Euro-IV vehicle standards for imported vehicles, regular emissions testing and certification for existing vehicles, diesel particulate filters, and establishing emissions-free vehicle zones in cultural and tourist sites. The action plan also outlines steps to manage construction activities to reduce dust resuspension, reducing industrial emissions, and improvements to household and agricultural waste management to decrease open burning. Additional short-term air pollution mitigation strategies may be implemented in case of air quality emergencies (i.e., when PM_2.5_ exceeds 300 μg m^–3^), such as limiting vehicle use to odd- or even-numbered license plates, closing factories, and prohibiting cargo trucks from entering the Kathmandu Valley. With garbage management (including stopping garbage fires) being the responsibility of local municipalities, source apportionment provides a clear expectation of the extent to which local action can improve air quality. Controlling garbage burning is the lowest hanging fruit (with no prior capital investment unlike vehicles and factories), and makes a significant difference during the polluted winter months. However, it alone will not achieve WHO air quality guidelines. Assessment of the efficacy of this plan necessitates continued and expanded air quality monitoring in the Kathmandu Valley, with source apportionment for pollutants that are targets for reductions.

This study advances the knowledge of primary sources and SOA formation in the Kathmandu Valley during the winter season that can inform strategies to combat unhealthy PM levels in this region. Abatement of road and construction dust is needed to reduce PM_10_ within WHO guidelines because the dust contribution to PM_10_ alone exceeds the WHO guideline value. Garbage burning, fossil fuel use, and much of biomass burning are anthropogenic and are theoretically controllable and should also be targets for ambient PM_2.5_ reductions. As the aromatic hydrocarbons are substantial contributors of SOC in the Kathmandu Valley and are associated with vehicles, garbage burning, and biomass burning, control of these primary anthropogenic sources would reduce primary emissions as well as SOA formation. Thus, a multipronged approach is needed to reduce ambient PM_2.5_ and PM_10_ in Kathmandu that addresses emissions from vehicles, garbage burning, biomass burning, and dust resuspension.

## Supporting Information Available

Calculation of the *o*-xylene contribution to the monoaromatic *f*_SOC_’ (Text S1); concentrations of PM_10_ mass, PM_2.5_ mass, and PM_2.5_ composition averaged to 23 h measurement (Table S1); 11 h average concentrations of metals in PM_2.5_ and PM_10_ at the Ratnapark site in Kathmandu (Table S2); intercorrelations (*r*) of PM_10_ mass and metal concentrations at the Ratnapark site (Table S3); 11 h average concentrations of select organic molecular markers for primary and secondary sources (Table S4); correlation (*r*) of NACs with levoglucosan and other NACs (Table S5); metal concentrations in PM_2.5_ and PM_10_ at the Ratnapark site (Figure S1); time series of SOA tracers from isoprene (Figure S2); time series of source contributions to PM_2.5_ organic carbon (Figure S3); (a) plot of TPB vs. Sb concentrations (ng m^–3^) in atmospheric PM_2.5_ at the Ratnapark site and ratios of Sb-to-TPB in garbage burning source samples during the NAMaSTE field campaign in 2015 in Nepal and (b) ambient measurements after logarithmic transformation (Figure S4); sensitivity of garbage burning contribution to garbage burning source profiles in CMB modeling (Figure S5); model diagnostics for the CMB source apportionment (Figure S6); sensitivity of biomass burning contribution to biomass burning source profiles in the CMB modeling (Figure S7); hopanes and other fossil fuel tracers across the Kathmandu Valley and comparison with prior studies (Figure S8); and sensitivity of the primary source contributions to the natural gas combustion source profile in the apportionment of PM_2.5_ OC using CMB modeling (Figure S9) (PDF)
