# Supplementary material for: Wintertime Air Quality across the Kathmandu Valley, Nepal: Concentration, Composition, and Sources of Fine and Coarse Particulate Matter
Source: ACS Earth Space Chem. 2022 Dec 6;6(12):2955–71. doi: 10.1021/acsearthspacechem.2c00243 (PMC9761783; doi:10.1021/acsearthspacechem.2c00243)
Supplement: Supplementary file 1 — sp2c00243_si_001.pdf [file sp2c00243_si_001.pdf]

SUPPORTING INFORMATION

Wintertime air quality across the Kathmandu Valley, Nepal: concentration, composition, and sources of fine and coarse particulate matter

Md. Robiul Islam[1], Tianyi Li[1], Khadak Mahata[2], Nita Khanal[2], Benjamin Werden[3a], Michael R. Giordano[4], Praveen Siva Puppala[5], Narayan Babu Dhital[6], Anobha Gurung[7], Eri Saikawa[8], Arnico K. Panday[9], Robert J. Yokelson[10], Peter F. DeCarlo[3,11], Elizabeth. A. Stone[1, 12*]

[1]University of Iowa, Department of Chemistry, Iowa City, Iowa 52242, USA
[2]Alpine Consultancy, Kathmandu 44600, Nepal
[3]Drexel University, Department of Civil, Architectural, and Environmental Engineering, Philadelphia, PA 19104, USA
[4] Université Paris-Est Cretéil and Université de Paris, CNRS, LISA, Créteil 94000, France
[5]International Centre for Integrated Mountain Development (ICIMOD), Khumaltar, Lalitpur 44700, Nepal
[6]Tribhuvan University, Patan Multiple Campus, Department of Environmental Science, Lalitpur 44700, Nepal
[7]Clean Cooking Alliance, Washington DC 20006, USA
[8]Emory University, Department of Environmental Sciences, Atlanta, Georgia 30322, USA
[9]Institute for Integrated Development Studies (IIDS), Kathmandu 44600, Nepal
[10]University of Montana, Department of Chemistry, Missoula, Montana 59812, USA
[11]Johns Hopkins University, Department of Environmental Health and Engineering, Baltimore, Maryland 21218
[12]University of Iowa, Department of Chemical and Biochemical Engineering, Iowa City, Iowa 52242, USA
[a]Now at: Aerodyne Research Inc, Center for Aerosol and Cloud Chemistry, Billerica, Massachusetts, 01821, USA

*Corresponding author - Email address: betsy-stone@uiowa.edu (E. A. Stone)

Contents of this file

Text S1- Calculation of the o-xylene contribution to the monoaromatic f_{SOC}'

Table S1 - Concentrations (µg m^{-3}) of PM_{10} mass; $PM_{2.5}$ mass; and $PM_{2.5}$ composition, including OC and EC across the Kathmandu Valley during winter of 2018 averaged to 23 h measurement for comparison to WHO guideline values that are assessed on a 24 h basis

Table S2 - 11 h average concentrations (ng m^{-3}) of metals in $PM_{2.5}$ and PM_{10} at the Ratnapark site in Kathmandu during January 18-27, 2018

Table S3 - Intercorrelations (r) of PM_{10} mass and metal concentrations at the Ratnapark site during January 18 to 27, 2018

Table S4 - 11 h average concentrations (ng m^{-3}) of select organic molecular markers for primary and secondary sources across the Kathmandu Valley, Nepal during winter 2018

Table S5 - Correlation (r) of NACs with levoglucosan and other NACs

Figure S1 - Metal concentrations in $PM_{2.5}$ and PM_{10} measured from January 18 to 27, 2018 at the Ratnapark site

Figure S2 - Time series of SOA tracers from isoprene

Figure S3 - Time series of source contributions to $PM_{2.5}$ organic carbon

Figure S4 - (a) A plot of TPB vs. Sb concentrations (ng m^{-3}) in atmospheric $PM_{2.5}$ at the Ratnapark site and ratios of Sb-to-TPB in garbage burning source samples during NAMaSTE field campaign in 2015 in Nepal. (b) Ambient measurements after log transformation

Figure S5 - Sensitivity of garbage burning contribution to garbage burning source profiles in the CMB modeling

Figure S6 - Model diagnostics for the CMB source apportionment across the Kathmandu Valley

Figure S7 – Sensitivity of biomass burning contribution to biomass burning source profiles in the CMB modeling

Figure S8 - Hopanes and other fossil fuel tracers across the Kathmandu Valley and comparison with prior studies

Figure S9 - Sensitivity of the primary source contributions to the natural gas combustion source profile in the apportionment of $PM_{2.5}$ OC using CMB modeling

SI section S1: Estimation of the o-xylene contribution to the monoaromatic f_{SOC}'

The portion of ambient phthalic acid estimated to form from o-xylene (x), rather than diaromatic VOC, was determined as the product of SOC from monoaromatic VOCs (μg m^{-3}), relative contribution of o-xylene to the monoaromatic f_{SOC}' (calculated to be 22.4%), and the o-xylene f_{SOC} for phthalic acid (0.045 ± 0.004).[1] The relative contribution of o-xylene to the monoaromatic f_{SOC}' (y) was calculated by equation (S1):

$$y = \frac{f_{SOC,x} Y_{SOC,x} [C_x]}{\sum_{i=1}^{n} f_{SOC,i} Y_{SOC,i} [C_i]} \ldots\ldots\ldots\ldots\ldots\ldots\ldots\ldots\ldots\ldots\ldots\ldots\ldots\ldots\ldots\ldots\ldots\ldots\ldots\ldots\ldots (S1)$$

Here, x represents o-xylene, i represents individual monoaromatic VOCs, and f_{SOC} represents tracer-to-SOC ratio, and Y_{SOC} represents SOC yield from VOC precursors in chamber experiments presented in Al-Naiema et al.[1]

Table S1. Concentrations (μg m^{-3}) of PM_{10} mass; $PM_{2.5}$ mass; and $PM_{2.5}$ composition, including OC and EC across the Kathmandu Valley during winter of 2018. The nighttime and daytime data from each date are averaged to 23 h measurement periods for comparison to WHO 2021 guideline values[2] and national ambient air quality standard (NAAQS) in Nepal[3] that are assessed on a 24 h basis. Data are shown as mean (standard deviation).

Locations	Dhulikhel	Ratnapark	Lalitpur	WHO guideline values	NAAQS, Nepal
**Number of 11h samples**	15	19	19	-	-
**PM_{10} mass** (μg m^{-3})	102.9 (25.4)	256.8 (41.3)	233.7 (61.6)	45	120
**$PM_{2.5}$ mass** (μg m^{-3})	54.8 (11.7)	120.6 (23.6)	129.5 (26.0)	15	40
**$PM_{2.5}$ OC** (μg m^{-3})	15.6 (3.9)	35.6 (4.8)	35.6 (6.8)	-	
**$PM_{2.5}$ EC** (μg m^{-3})	5.1 (1.2)	16.7 (3.5)	12.2 (2.5)	-	

Table S2. 11 h average concentrations (ng m^{-3}) of metals in $PM_{2.5}$ and PM_{10} at the Ratnapark site in Kathmandu during January 18-27, 2018.

Metals (ng m^{-3})	**Concentrations in $PM_{2.5}$** (n=19)			**Concentrations in PM_{10}** (n=19)		
	Range	Mean (SD)	Median	Range	Mean (SD)	Median
Magnesium	84-332	250 (60)	264	692-2711	1790 (546)	1859
Aluminum	494-1838	1361 (333)	1400	3720-16470	9502 (3315)	10038
Potassium	421-1675	1242 (300)	1356	155-6389	3334 (1805)	3556
Calcium	62-298	206 (59)	208	500-2086	1403 (436)	1426
Titanium	22.6-66.3	50.2 (10.8)	51.6	134-571	345 (116)	358
Vanadium	0.75-3.1	2.2 (0.5)	2.3	4.9-20.5	12.8 (4.2)	13.9
Chromium	<0.08-35.0	6.6 (10.5)	2.5	0.42-32.3	14.8 (9.5)	11.9
Manganese	6.9-29.9	19.2 (5.1)	18.8	37.8-146	102 (31)	107
Iron	357-1257	952 (229)	1004	2161-9225	6157 (1999)	6524
Cobalt	0.24-1.1	0.55 (0.21)	0.54	0.78-3.4	2.3 (0.7)	2.4
Nickel	<0.001-4.6	1.6 (1.7)	1.3	<0.001-6.7	2.6 (2.1)	1.9
Copper	0.19-144	26.8 (31.9)	22.2	4.6-178	42.6 (38.7)	37.7
Zinc	<1.0-580	299 (161)	310	62.3-666	306 (195)	263
Arsenic	0.57-2.7	1.7 (0.5)	1.8	0.71-4.5	2.8 (0.9)	3.1
Selenium	<0.01-9.7	4.5 (2.8)	4.2	<0.01-6.9	3.1 (2.0)	3.0
Strontium	0.60-3.2	2.4 (0.6)	2.6	5.8-23.6	14.6 (4.3)	15.5
Molybdenum	0.07-1.03	0.52 (0.24)	0.45	0.21-1.32	0.81 (0.28)	0.85
Cadmium	0.27-18.2	4.4 (4.2)	3.2	0.78-19.0	4.7 (4.1)	3.9
Antimony	2.4-54.5	13.4 (14.2)	9.2	1.2-64.0	14.7 (15.9)	10.6
Cesium	0.08-0.38	0.28 (0.07)	0.30	0.57-2.3	1.5 (0.5)	1.6
Barium	6.6-27.2	18.8 (5.2)	19.1	37.4-172	105 (38)	109
Cerium	0.52-1.9	1.5 (0.4)	1.5	4.2-16.5	11.7 (3.6)	12.6
Lead	12.5-123	47.7 (27.0)	39.9	10.1-146	58.1 (31.1)	49.9

Table S3. Intercorrelations (r) of PM_{10} mass and metal concentrations at the Ratnapark site during January 18 to 27, 2018.

	PM_{10}	Mg	Al	K	Ca	Ti	V	Mn	Fe	Co	Cu	Zn	As	Sr	Mo	Sb	Cs	Ba	Ce
Mg	0.95																		
Al	0.88	0.97																	
K	0.90	0.96	0.98																
Ca	0.95	0.98	0.93	0.93															
Ti	0.91	0.98	0.99	0.98	0.94														
V	0.93	0.98	0.98	0.98	0.95	0.99													
Mn	0.97	0.99	0.94	0.95	0.98	0.96	0.97												
Fe	0.96	>0.99	0.96	0.97	0.98	0.98	0.98	>0.99											
Co	0.96	0.99	0.94	0.95	0.98	0.96	0.97	0.99	0.99										
Cu	0.46	0.41	0.34	0.38	0.45	0.32	0.34	0.41	0.41	0.42									
Zn	0.32	0.21	0.16	0.19	0.28	0.17	0.14	0.27	0.24	0.25	0.60								
As	0.94	0.89	0.79	0.82	0.92	0.82	0.84	0.92	0.90	0.91	0.64	0.45							
Sr	0.92	0.96	0.93	0.89	0.97	0.94	0.94	0.94	0.95	0.93	0.36	0.19	0.86						
Mo	0.69	0.62	0.55	0.50	0.65	0.58	0.57	0.63	0.62	0.63	0.35	0.51	0.61	0.67					
Sb	0.58	0.58	0.50	0.57	0.60	0.50	0.54	0.57	0.58	0.57	0.78	0.25	0.67	0.51	0.15				
Cs	0.96	0.99	0.95	0.95	0.97	0.97	0.98	0.99	0.99	0.98	0.33	0.19	0.87	0.95	0.63	0.52			
Ba	0.92	0.98	0.98	0.99	0.96	0.99	>0.99	0.97	0.98	0.97	0.36	0.17	0.85	0.93	0.54	0.56	0.97		
Ce	0.95	0.98	0.93	0.92	0.97	0.95	0.95	0.98	0.98	0.98	0.43	0.31	0.90	0.94	0.70	0.56	0.98	0.95	
Pb	0.77	0.72	0.66	0.71	0.75	0.65	0.68	0.73	0.74	0.72	0.79	0.48	0.83	0.67	0.52	0.78	0.70	0.70	0.73

Table S4: 11 h average concentrations (ng m^{-3}) of select organic molecular markers for primary and secondary sources across the Kathmandu Valley, Nepal during winter 2018.

	**Dhulikhel**			**Ratnapark**			**Lalitpur**		
Location	27.608 °N, 85.547 °E			27.706 °N, 85.316 °E			27.646 °N,85.324 °E		
Altitude	1600 m (a.s.l)			1300 m (a.s.l)			1300 m (a.s.l)		
Dates in 2018	January 7-14			January 18-27			February 1-10		
Number of samples	15			19			19		
Data type	Range (ng m^{-3})	Mean (SD) (ng m^{-3})	Median (ng m^{-3})	Range (ng m^{-3})	Mean (SD) (ng m^{-3})	Median (ng m^{-3})	Range (ng m^{-3})	Mean (SD) (ng m^{-3})	Median (ng m^{-3})
Levoglucosan	607-1302	944 (207)	938	1059-3533	2201 (757)	2057	843-2208	1398 (406)	1303
**Sterols**									
Cholesterol[a]	NR	NR	NR	0.64-9.1	3.9 (2.3)	3.9	0.32-10.2	2.4 (2.7)	1.5
Stigmasterol	BDL-4.00	1.07 (1.57)	0.11	2.8-12.6	7.0 (3.5)	6.1	1.9-16.6	4.6 (3.5)	3.4
β-Sitosterol[a]	BDL-29.8	NR	NR	7.2-47.3	23.8 (15.1)	19.7	5.8-57.6	16.0 (12.0)	13.1
Campesterol	0.13-1.56	0.84 (0.50)	0.83	1.8-13.5	6.75 (4.43)	5.15	0.62-16.9	3.6 (3.7)	2.3
Coprostanol[a]	BDL-0.01	NR	NR	0.23-0.70	0.47 (0.14)	0.42	BDL-2.0	NR	NR
Stigmastanol	0.31-1.51	0.87 (0.39)	0.84	1.2-4.6	2.79 (1.02)	2.88	1.5-6.2	2.9 (1.2)	2.5
**Nitro-aromatic compounds (NACs)**									
4-Nitrophenol (4NP)	9.5-48.8	28.4 (12.8)	25.0	18.5-48.7	26.8 (6.6)	25.6	17.8-47.0	30.9 (8.3)	28.0
4-methyl-3-nitrophenol (4M-3NP)	BDL	BDL	BDL	BDL	BDL	BDL	1.3-8.2	2.8 (1.5)	2.5
2-Methyl-4-nitrophenol (2M-4NP)	5.7-16.9	12.0 (3.5)	11.1	5.7-19.5	9.80 (3.62)	9.32	4.4-17.0	9.2 (3.5)	8.0
4-Nitroguaiacol (4NG)	6.6-41.0	20.0 (12.4)	17.8	1.9-8.6	4.64 (2.09)	3.97	2.5-35.4	9.1 (8.6)	5.9
4-Hydroxy-3-nitrobenzyl alcohol (4H-3NB)	1.87-2.48	2.19 (0.18)	2.18	1.5-6.2	3.60 (1.42)	3.62	1.5-3.7	2.4 (0.7)	2.2
4-Nitrocatechol (4NC)	13.8-70.3	40.9 (19.7)	37.4	15.1-488	206 (122)	224	50.1-381	152 (90)	124
4-Methyl-5-nitrocatechol (4M-5NC)	3.4-18.9	10.2 (5.1)	9.7	9.3-181	76.4 (49.6)	56.4	21.7-173	60.6 (45.7)	43.3
3-Methyl-6-nitrocatechol (3M-6NC)	0.17-1.54	0.77 (0.44)	0.67	1.5-12.8	5.86 (3.45)	4.80	2.1-9.5	4.4 (2.2)	3.8

Table S4 continued

	Dhulikhel			Ratnapark			Lalitpur		
5-Nitrosalicylic acid (5NSA)	5.43-9.25	6.92 (1.22)	6.87	2.2-20.2	6.26 (3.87)	5.96	2.6-11.2	5.8 (2.5)	6.2
**Polycyclic aromatic hydrocarbons (PAHs)**									
Fluoranthene	2.1-4.7	3.4 (0.79)	3.3	2.9-12.0	6.0 (2.0)	5.4	3.5-11.5	5.8 (2.2)	5.2
Pyrene	2.0-4.2	3.1 (0.7)	3.0	3.3-12.6	6.6 (2.2)	5.9	3.5-11.9	5.9 (2.3)	5.2
Methylfluoranthene	0.62-1.2	0.87 (0.19)	0.82	1.1-4.4	2.1 (0.8)	1.8	1.0-3.9	1.9 (0.8)	1.7
Benzo(ghi)fluoranthene	2.3-4.4	3.2 (0.8)	3.0	4.3-17.7	9.1 (3.2)	7.9	3.9-16.8	7.5 (3.3)	6.8
Benz(a)anthracene	0.99-2.0	1.4 (0.3)	1.4	1.8-11.7	5.1 (2.3)	4.5	2.3-9.2	4.3 (2.0)	4.3
Chrysene	1.8-4.2	2.9 (0.8)	2.8	3.3-15.8	7.9 (2.7)	7.5	4.1-17.2	7.7 (3.3)	6.7
1-Methylchrysene	0.10-0.43	0.24 (0.10)	0.24	0.33-1.3	0.78 (0.26)	0.74	0.33-1.7	0.74 (0.38)	0.71
Retene	0.17-0.80	0.39 (0.17)	0.36	0.80-4.8	2.5 (1.4)	2.4	0.20-4.9	1.7 (1.2)	1.5
Benzo(b)fluoranthene	2.1-4.4	3.1 (0.8)	3.2	3.7-14.2	8.1 (2.4)	8.5	4.6-17.9	8.2 (3.3)	7.4
Benzo(k)fluoranthene	1.8-4.3	2.8 (0.8)	2.8	3.3-13.7	7.9 (2.4)	8.1	4.2-17.7	8.0 (3.6)	7.2
Benzo(j)fluoranthene[b]	0.39-0.88	0.61 (0.15)	0.59	0.72-5.0	2.2 (1.0)	1.9	0.83-4.2	1.9 (0.9)	1.6
Benzo(e)pyrene	1.3-2.9	2.1 (0.6)	2.2	2.5-10.5	6.2 (1.8)	6.2	2.9-13.6	6.0 (2.6)	5.5
Benzo(a)pyrene	1.4-2.8	2.1 (0.5)	2.0	2.5-12.8	6.7 (2.6)	6.3	2.7-13.4	5.9 (2.8)	4.9
Perylene	0.26-0.53	0.38 (0.08)	0.38	0.47-3.0	1.3 (0.6)	1.2	0.33-2.5	0.98 (0.53)	0.77
Indeno(1,2,3-cd)pyrene	1.9-4.0	2.8 (0.7)	2.9	3.3-13.8	7.3 (2.3)	7.4	3.7-17.9	7.5 (3.2)	6.6
Dibenz(ah)anthracene	0.27-3.4	1.5 (1.1)	1.8	0.49-1.5	1.0 (0.3)	1.1	0.36-2.1	0.95 (0.44)	0.84
Benzo(ghi)perylene	0.02-3.1	1.1 (1.3)	0.28	3.7-12.3	7.7 (2.0)	7.8	3.0-16.7	6.9 (3.3)	6.2
Picene	0.37-1.2	0.69 (0.25)	0.70	0.78-3.2	1.8 (0.6)	1.9	1.2-4.2	2.1 (0.8)	1.9
1,3,5-Triphenylbenzene	0.37-0.97	0.60 (0.20)	0.53	1.1-17.3	4.5 (3.6)	4.2	1.2-10.1	3.7 (2.8)	2.7
**∑ PAHs**	22.9-45.7	33.3 (8.1)	33.1	41.1-174	94.9 (31.2)	90.5	45.7-190	87.6 (37.0)	78.7
**Hopanes**									
17α(H)-21β(H)-Hopane	0.13-0.87	0.36 (0.25)	0.26	1.0-2.5	1.7 (0.5)	1.6	0.27-2.5	0.74 (0.50)	0.57
17β(H)-21α(H)-30-Norhopane	0.10-0.44	0.27 (0.10)	0.23	1.3-2.9	2.1 (0.50)	2.2	0.30-2.7	0.82 (0.55)	0.71
17α(H)-22,29,30-Trisnorhopane	0.03-0.24	0.11 (0.06)	0.11	0.24-0.94	0.55 (0.20)	0.54	0.09-1.0	0.32 (0.25)	0.25
**∑Hopanes**	0.26-1.39	0.74 (0.37)	0.68	2.8-6.4	4.4 (1.1)	4.5	0.80-6.2	1.9 (1.3)	1.6

Table S4 continued

	Dhulikhel			Ratnapark			Lalitpur		
**Aromatic SOA tracers**									
DHOPA	3.6-8.1	5.6 (1.3)	5.5	3.5-9.1	6.6 (1.6)	6.7	4.2-11.2	7.7 (1.9)	7.7
Phthalic acid	39.3-96.5	65.4 (15.1)	61.9	61.2-151.9	95.6 (22.1)	92.2	45.4-168	90.4 (29.5)	81.6
4-Methylphthalic acid	5.9-15.0	9.7 (2.7)	9.14	3.6-16.2	9.2 (3.8)	7.68	5.5-19.1	9.4 (3.4)	8.7
Terephthalic acid	5.5-12.2	9.1 (2.0)	8.89	7.6-25.8	16.5 (5.4)	15.3	3.7-23.2	13.2 (6.9)	12.0
Isophthalic acid	108-307	176 (60)	162	270-1034	590 (193)	575	193-1984	703 (565)	478
**Isoprene, monoterpene, and sesquiterpene SOA tracers**									
2-Methylglyceric acid	1.7-4.2	3.0 (0.7)	3.0	6.7-12.6	10.3 (2.1)	11.2	7.3-17.6	12.8 (2.9)	13.0
2-Methylthreitol[c]	1.9-4.3	3.3 (0.7)	3.4	2.2-6.4	3.9 (1.1)	4.17	3.9-10.8	7.0 (1.9)	6.8
2-Methylerythritol[c]	2.2-5.2	3.9 (0.9)	4.1	BDL-5.2	2.2 (1.4)	2.05	4.2-10.5	7.2 (1.9)	7.2
Cis-pinonic acid	BDL	BDL	BDL	0.05-3.4	1.4 (1.2)	1.5	0.08-4.7	1.6 (1.4)	1.3
3-Hydroxyglutaric acid[e]	9.9-37.1	24.6 (7.6)	24.9	7.8-105	41.2 (28.9)	29.7	2.0-77.3	34.4 (27.3)	25.1
3-Acetyladipic acid[d]	15.0-33.3	24.0 (5.9)	22.5	14.9-78.8	44.8 (21.0)	43.4	17.7-50.0	32.5 (9.0)	34.0
β-Caryophyllinic acid[d]	9.4-24.2	15.1 (3.4)	14.7	20.3-65.8	35.3 (11.1)	31.7	18.9-59.4	29.3 (10.8)	26.2

SD = Standard deviation; BDL = Below detection limit; [a]Low data coverage <40%, mean and median not reported (NR), [b]Semi-quantified using benzo(k)fluoranthene; [c]Semi-quantified using meso-erythritol calibration; [d]Semi-quantified using cis-pinonic acid calibration.

Table S5. Correlation (r) of NACs with levoglucosan and other NACs. Correlation (r) of brown carbon with NACs and levoglucosan is also shown for Ratnapark site. Correlations that are significant in 95% confidence interval (CI) are marked with one star (*) and that are significant in 99% CI are marked with two stars (**).

	**Levoglucosan**	**4NC**	**4M-5NC**	**3M-6NC**	**NSA**	**4NP**
**Dhulikhel**						
4NC	0.75**					
4M-5NC	0.69**	0.99**				
3M-6NC	0.80**	0.91**	0.88**			
NSA	0.72**	0.68**	0.70**	0.47		
4NP	0.50	0.58**	0.52	0.76**	-0.03	
2M-4NP	0.52	0.63**	0.57*	0.79**	0.02	0.98**
Brown carbon	0.64*	0.25	0.23	0.22	0.62*	0.12
**Ratnapark**						
4NC	0.81**					
4M-5NC	0.83**	0.96**				
3M-6NC	0.80**	0.91**	0.98**			
NSA	0.55*	0.35	0.22	0.17		
4NP	0.40	0.69**	0.70**	0.73**	0.03	
2M-4NP	0.62**	0.75**	0.64**	0.61**	0.65**	0.70**
Brown carbon	0.81**	0.68**	0.70**	0.66**	0.54*	0.56*
**Lalitpur**						
4NC	0.81**					
4M-5NC	0.82**	0.96**				
3M-6NC	0.87**	0.97**	0.98**			
NSA	0.05	0.05	0.02	0.11		
4NP	0.44	0.63**	0.73**	0.69**	-0.17	
2M-4NP	0.58**	0.82**	0.85**	0.83**	-0.09	0.90**

Figure S1. Metal concentrations in $PM_{2.5}$ and PM_{10} measured from January 18 to 27, 2018 at the Ratnapark site shown on a logarithmic scale. The boxes show the interquartile range and median, upper and lower bars show the range, and outliers are marked by dots. The metals are presented in order of the greatest to lowest mass difference for the measured species in PM_{10} relative to $PM_{2.5}$.

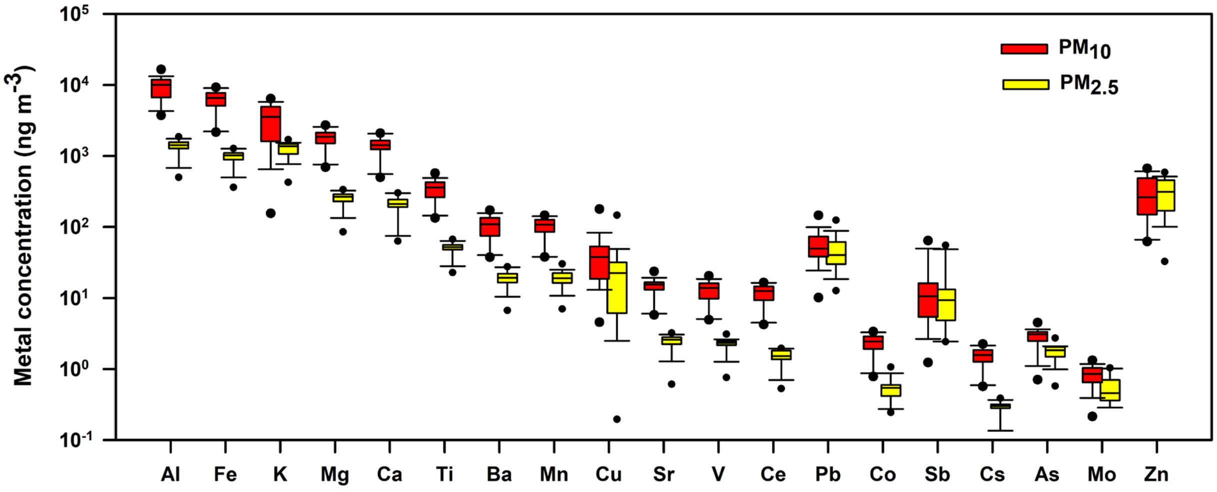

Figure S2. Time series of SOA tracers from isoprene. The ratio of 2-methyltetrols (2-methylthreitol and 2-methylerythritol) to 2-methylglyceric acid was 2.4, 1.8, and 1.1 for Dhulikhel, Ratnapark, and Lalitpur, respectively. These ratios showed an abundance of low-NO_x tracers 2-methyltetrols at the rural Dhulikhel site, which is consistent with the lower NO_x level at that location compared to urban and suburban in-Valley sites. Daytime samples are labeled as D and nighttime samples are labeled as N or dash (-). The night of February 8 is not reported (NR) due to unknown sampling time.

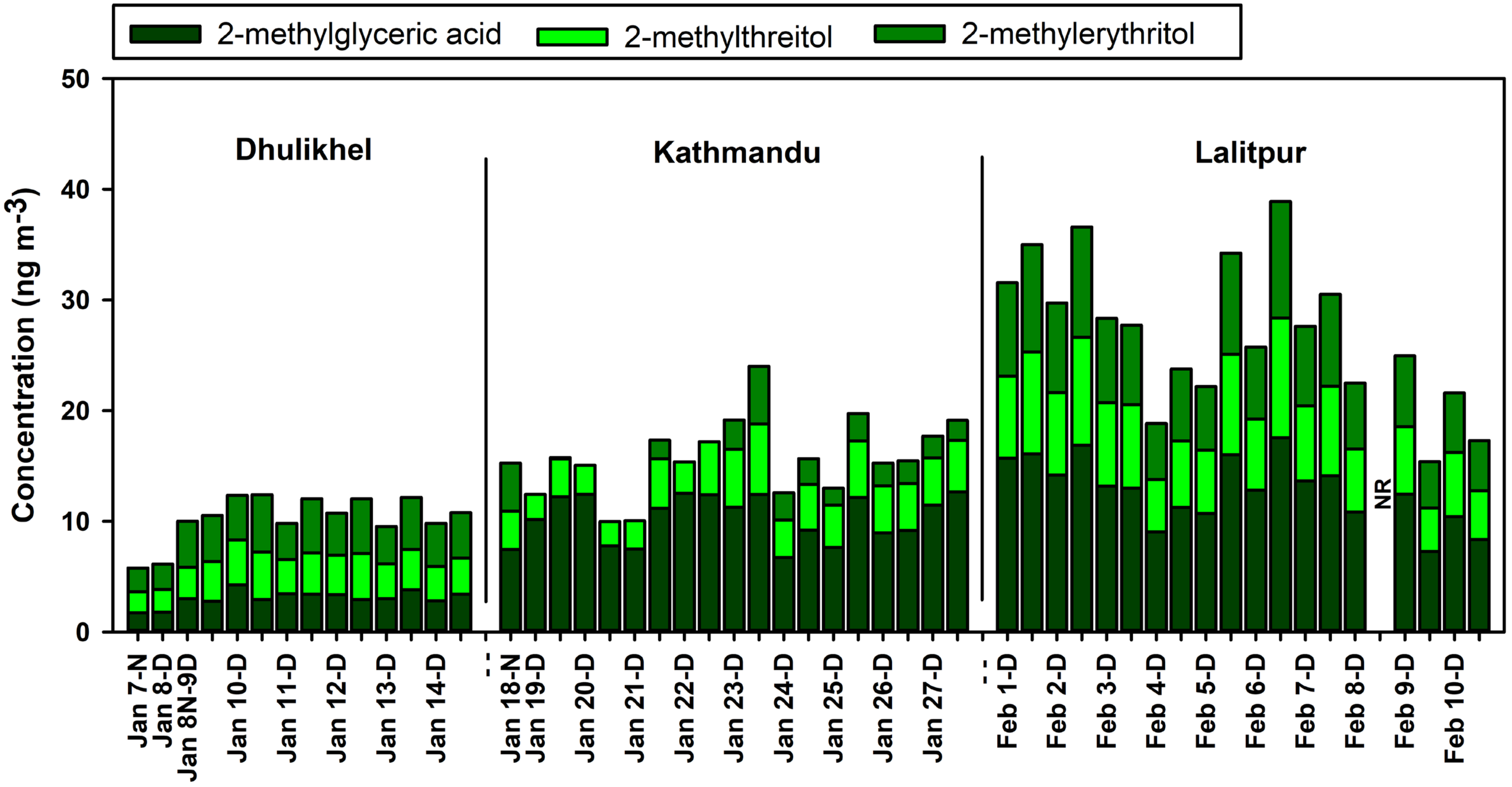

Figure S3. Time series of source contributions to $PM_{2.5}$ organic carbon. Daytime samples are labeled as D and nighttime samples are labeled as N or dash (-). The night of February 8 is not reported (NR) due to unknown sampling time.

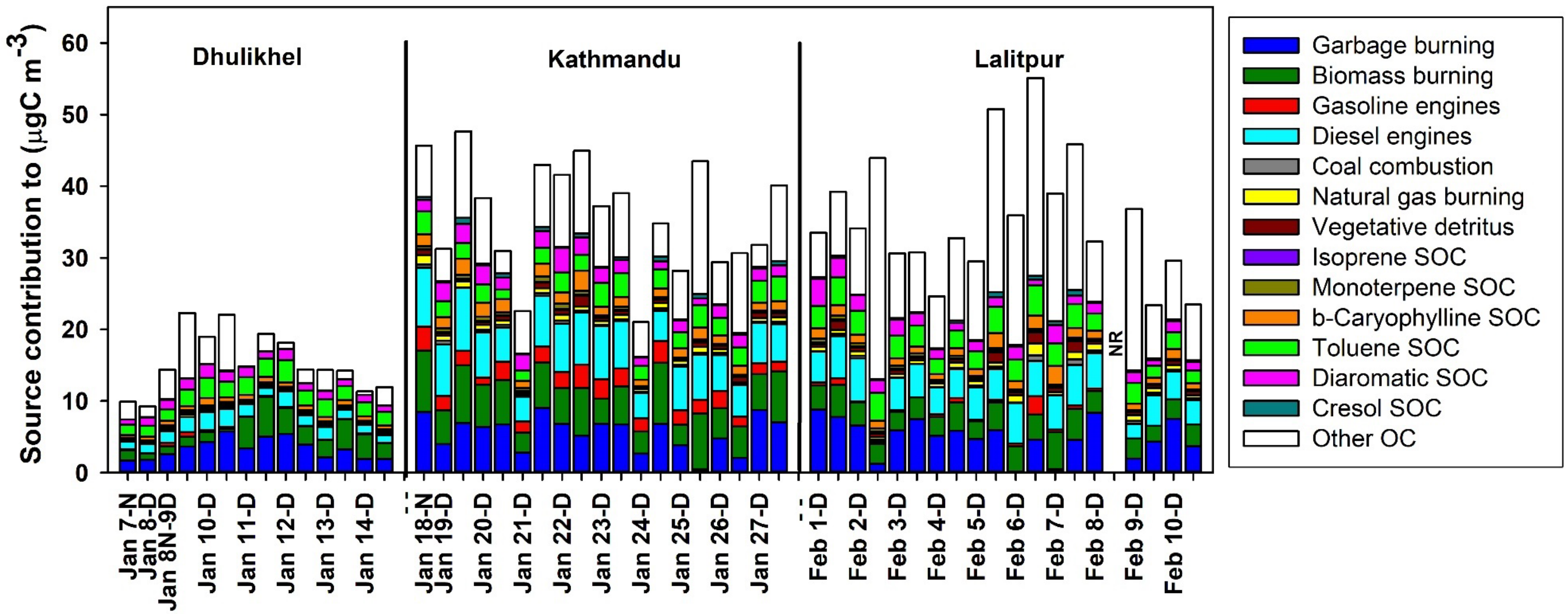

Figure S4. (a) A plot of TPB vs. Sb concentrations (ng m^{-3}) in atmospheric $PM_{2.5}$ at the Ratnapark site (shown as black dots) and ratios of Sb-to-TPB in garbage burning source samples during NAMaSTE field campaign in 2015 in Nepal (shown as solid lines). (b) Ambient measurements after log transformation.

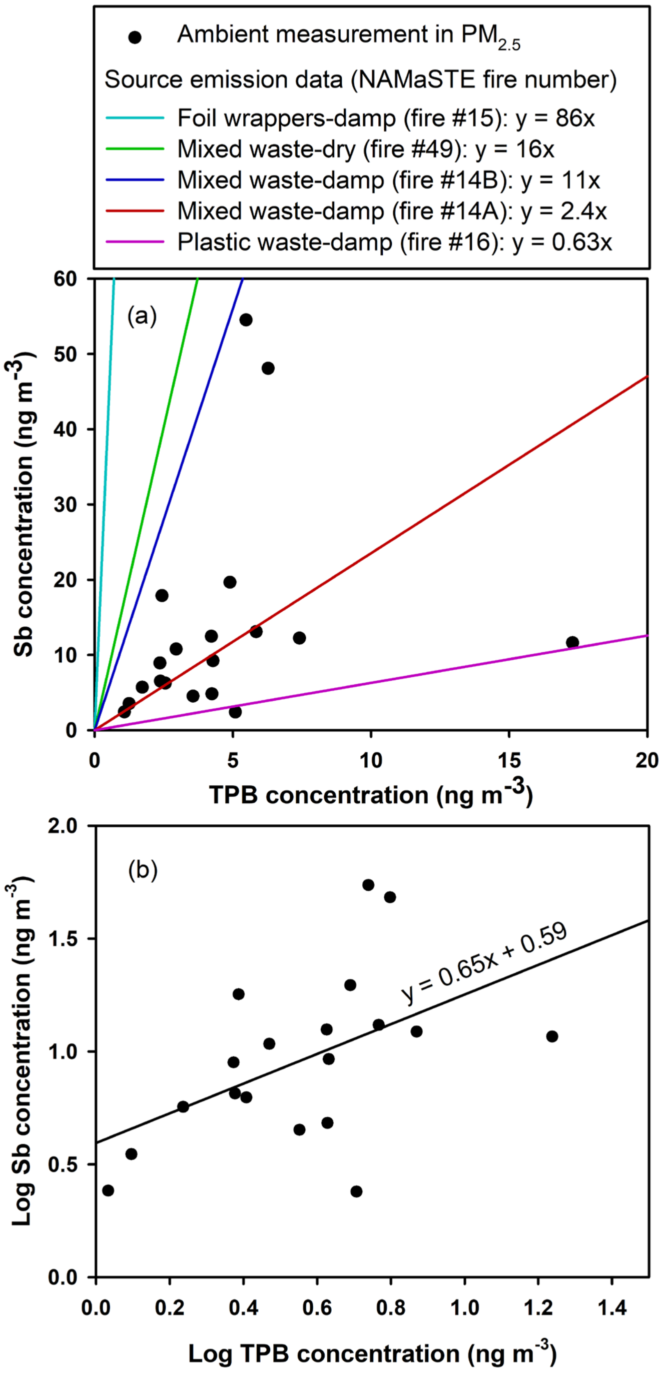

Figure S5. Sensitivity of the garbage burning contribution to garbage burning source profiles in the CMB modeling. OC apportioned to garbage burning increased by 7%, 56%, and 72% in Dhulikhel, Ratnapark, and Lalitpur, respectively, when mixed garbage burning profile A was used instead of mixed garbage burning profile B both drawn from fire number 14 in Jayarathne et al.[4]

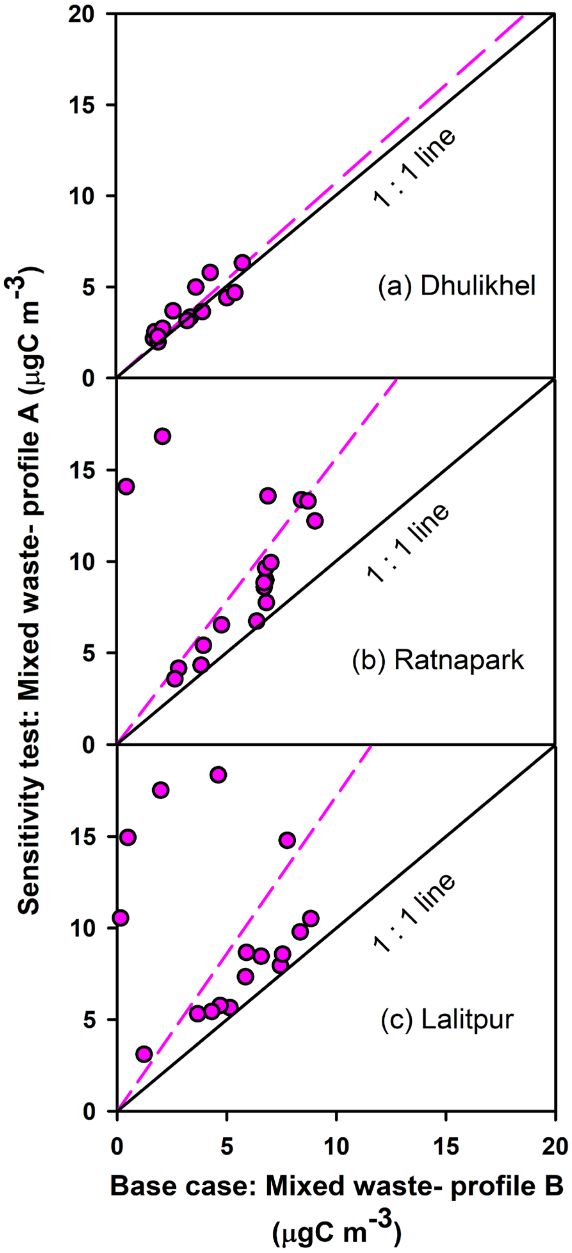

Figure S6. Model diagnostics for the CMB source apportionment across the Kathmandu Valley for 11h $PM_{2.5}$ samples. (a) The R^2 value for the measured and calculated concentrations ranged 0.75-0.88. (b) The χ^2 values represent the difference between calculated and measured species concentrations and ranged 3-7.

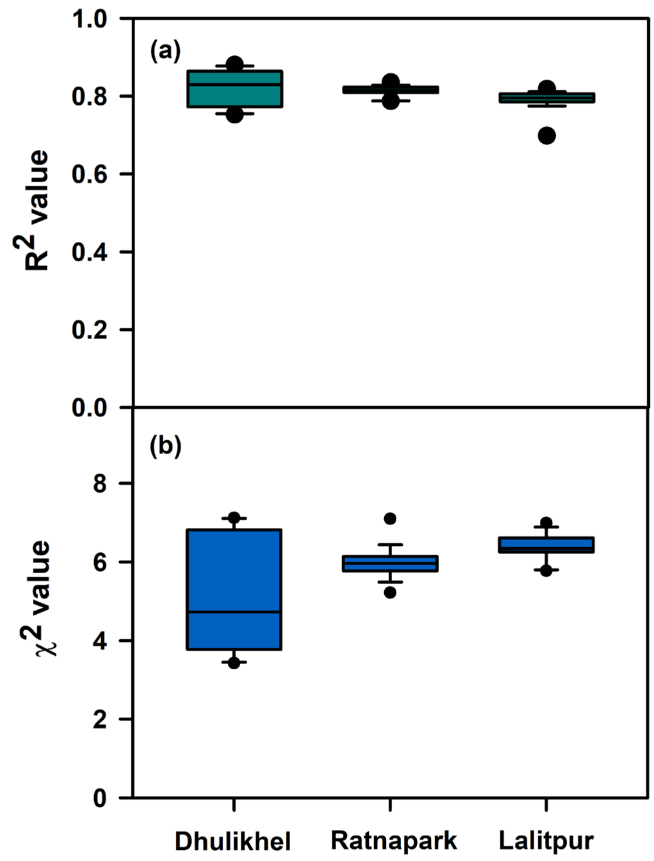

Figure S7. Sensitivity of the biomass burning contribution to biomass burning source profiles in the CMB modeling. Changing the biomass profile from the base case profile (Mud stove fueled with wood for Dhulikhel and Open fire with twigs and dung for Ratnapark and Lalitpur) to other biomass profiles changed the OC apportioned to biomass by factors of 0.71-0.97 at Dhulikhel (a), 0.30-0.63 at Ratnapark (b), and 0.10-0.63 at Lalitpur (c).

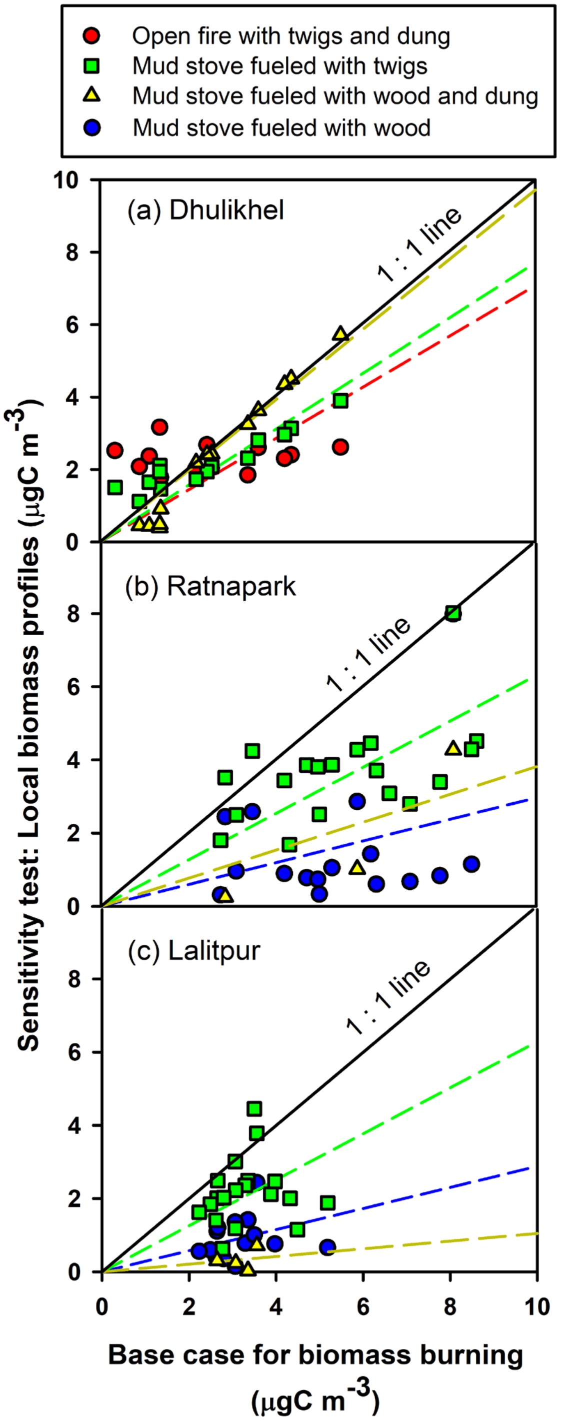

Figure S8. Comparison of concentrations of hopanes (a fossil fuel tracer), OC-to-EC ratios, and polycyclic aromatic hydrocarbons (products of combustion) in this and select prior studies in Nepal: at Bode in the Kathmandu Valley[5] and Lumbini[6]

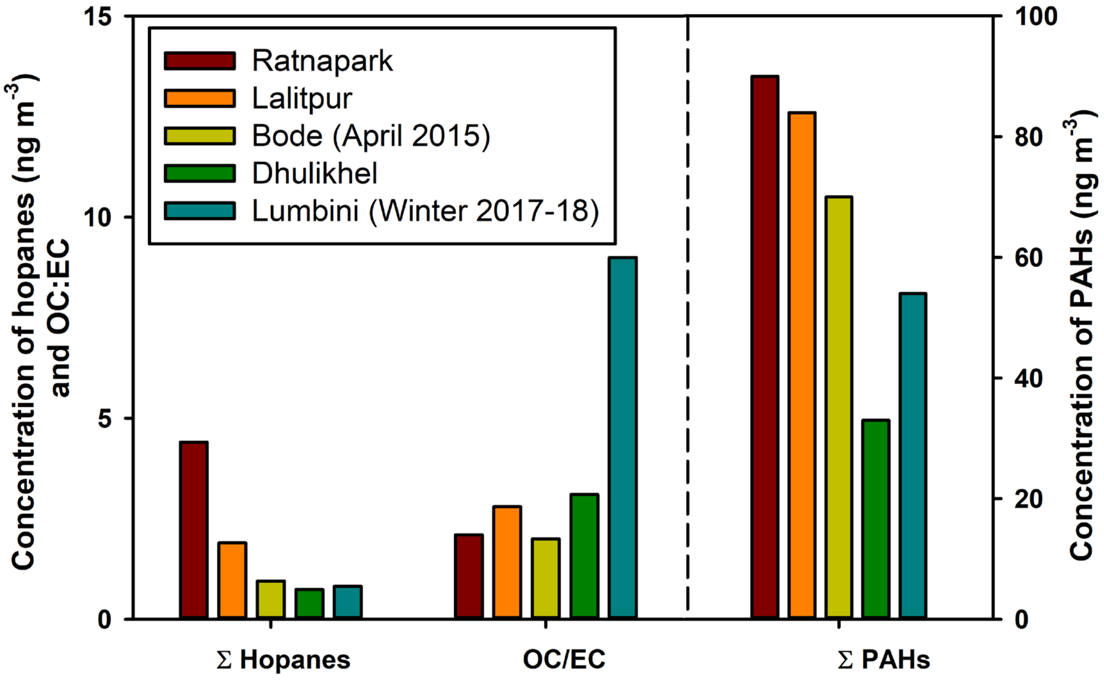

Figure S9. Sensitivity of the primary source contributions to the natural gas combustion source profile in the apportionment of $PM_{2.5}$ OC using CMB modeling. When the natural gas profile was excluded from the source apportionment modeling, the apportioned OC changed by factors of 1.3-1.7 for garbage burning (a), 0.96-1.1 for biomass burning (b), 0.50-0.77 for gasoline engines (c), 0.98-1.0 for diesel engines (d), 1.2-2.1 for coal combustion (e), and 0.71-0.87 for vegetative detritus (f). These results indicated that adding the natural gas profile decreased the gasoline engine source contribution, while increasing those of garbage burning and coal combustion.

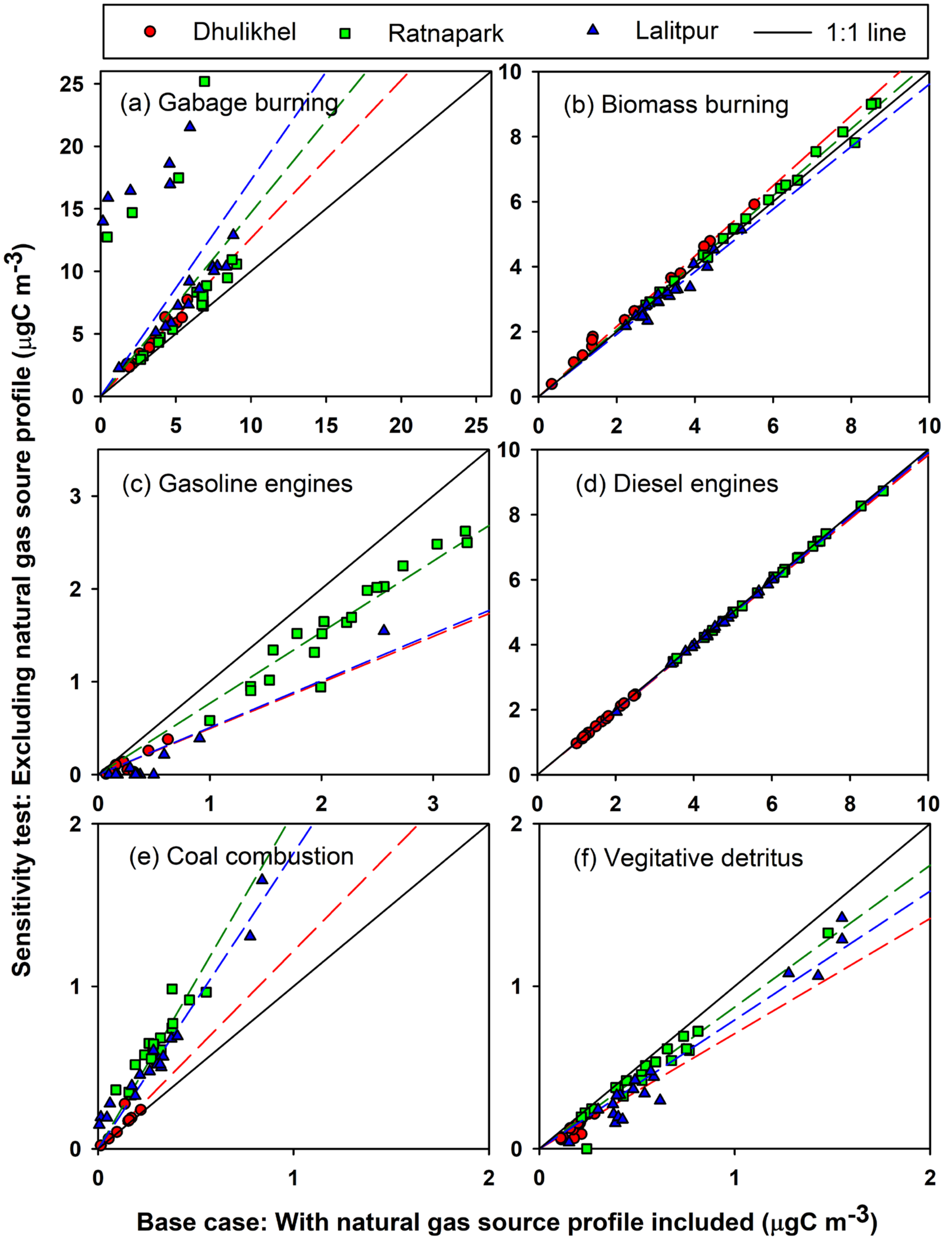

Works Cited

(1) Al-Naiema, I. M.; Offenberg, J. H.; Madler, C. J.; Lewandowski, M.; Kettler, J.; Fang, T.; Stone, E. A., Secondary organic aerosols from aromatic hydrocarbons and their contribution to fine particulate matter in Atlanta, Georgia. *Atmos. Environ.* **2020,** *223*, 117227.

(2) WHO, "WHO global air quality guidelines: particulate matter (PM2.5 and PM10), ozone, nitrogen dioxide, sulfur dioxide and carbon monoxide" Retrieved from-https://www.who.int/publications/i/item/9789240034228, Last accessed-11 July 2022. World Health Organization: 2021.

(3) NAAQS, "Ambient Air Quality monitoring program" Retrieved from-http://doenv.gov.np/public/uploads/Pdffile/Aqms%20report%202017-1-52472-45751.pdf, Last accessed-06 August 2022. Government of Nepal: 2017.

(4) Jayarathne, T.; Stockwell, C. E.; Bhave, P. V.; Praveen, P. S.; Rathnayake, C. M.; Islam, M. R.; Panday, A. K.; Adhikari, S.; Maharjan, R.; Goetz, J. D.; DeCarlo, P. F.; Saikawa, E.; Yokelson, R. J.; Stone, E. A., Nepal Ambient Monitoring and Source Testing Experiment (NAMaSTE): emissions of particulate matter from wood- and dung-fueled cooking fires, garbage and crop residue burning, brick kilns, and other sources. *Atmos. Chem. Phys.* **2018,** *18* (3), 2259-2286.

(5) Islam, M. R.; Jayarathne, T.; Simpson, I. J.; Werden, B.; Maben, J.; Gilbert, A.; Praveen, P. S.; Adhikari, S.; Panday, A. K.; Rupakheti, M.; Blake, D. R.; Yokelson, R. J.; DeCarlo, P. F.; Keene, W. C.; Stone, E. A., Ambient air quality in the Kathmandu Valley, Nepal, during the pre-monsoon: concentrations and sources of particulate matter and trace gases. *Atmos. Chem. Phys.* **2020,** *20* (5), 2927-2951.

(6) Islam, M. R.; Li, T.; Mahata, K.; Khanal, N.; Werden, B.; Giordano, M. R.; Praveen, P. S.; Dhital, N. B.; Gurung, A.; Panday, A. K.; Joshi, I. B.; Poudel, S. P.; Wang, Y.; Saikawa, E.; Yokelson, R. J.; DeCarlo, P. F.; Stone, E. A., Wintertime Air Quality in Lumbini, Nepal: Sources of Fine Particle Organic Carbon. *ACS Earth and Space Chemistry* **2021,** *5* (2), 226-238.
